# An epigenome-wide association study of Alzheimer's disease blood highlights robust DNA hypermethylation in the *HOXB6* gene

**DOI:** 10.1016/j.neurobiolaging.2020.06.023

**Published:** 2020-11

**Authors:** Janou A.Y. Roubroeks, Adam R. Smith, Rebecca G. Smith, Ehsan Pishva, Zina Ibrahim, Martina Sattlecker, Eilis J. Hannon, Iwona Kłoszewska, Patrizia Mecocci, Hilkka Soininen, Magda Tsolaki, Bruno Vellas, Lars-Olof Wahlund, Dag Aarsland, Petroula Proitsi, Angela Hodges, Simon Lovestone, Stephen J. Newhouse, Richard J.B. Dobson, Jonathan Mill, Daniël L.A. van den Hove, Katie Lunnon

**Affiliations:** a College of Medicine and Health, University of Exeter, Exeter, UK; b School for Mental Health and Neuroscience (MHeNS), Department of Psychiatry and Neuropsychology, Maastricht University, Maastricht, the Netherlands; c Department of Biostatistics and Health Informatics, Institute of Psychiatry, Psychology and Neuroscience (IOPPN), King's College London, London, UK; d Farr Institute of Health Informatics Research, University College London, London, UK; e Medical University of Lodz, Lodz, Poland; f Institute of Gerontology and Geriatrics, University of Perugia, Perugia, Italy; g Institute of Clinical Medicine, Neurology, University of Eastern Finland, Kuopio, Finland; h Neurocenter, Neurology, Kuopio University Hospital, Kuopio, Finland; i 1st Department of Neurology, Memory and Dementia Unit, Aristotle University of Thessaloniki, Thessaloniki, Greece; j INSERM U 558, University of Toulouse, Toulouse, France; k NVS Department, Section for Clinical Geriatrics, Karolinska Institutet, Stockholm, Sweden; l King's Health Partners Centre for Neurodegeneration Research, Institute of Psychiatry, Psychology and Neuroscience, King's College London, London, UK; m Centre for Age-Related Diseases, Stavanger University Hospital, Stavanger, Norway; n NIHR Biomedical Research Centre for Mental Health at South London and Maudsley NHS Foundation Trust and Institute of Psychiatry, King's College London, London, UK; o Department of Psychiatry, Warneford Hospital, University of Oxford, Oxford, UK; p Current Affiliation at Janssen-Cilag UK; q Department of Psychiatry, Psychosomatics and Psychotherapy, University of Würzburg, Würzburg, Germany

**Keywords:** Alzheimer's disease (AD), Biomarker, Blood, DNA methylation, *HOXB6*, Mild cognitive impairment (MCI)

## Abstract

A growing number of epigenome-wide association studies have demonstrated a role for DNA methylation in the brain in Alzheimer's disease. With the aim of exploring peripheral biomarker potential, we have examined DNA methylation patterns in whole blood collected from 284 individuals in the AddNeuroMed study, which included 89 nondemented controls, 86 patients with Alzheimer's disease, and 109 individuals with mild cognitive impairment, including 38 individuals who progressed to Alzheimer's disease within 1 year. We identified significant differentially methylated regions, including 12 adjacent hypermethylated probes in the *HOXB6* gene in Alzheimer's disease, which we validated using pyrosequencing. Using weighted gene correlation network analysis, we identified comethylated modules of genes that were associated with key variables such as *APOE* genotype and diagnosis. In summary, this study represents the first large-scale epigenome-wide association study of Alzheimer's disease and mild cognitive impairment using blood. We highlight the differences in various loci and pathways in early disease, suggesting that these patterns relate to cognitive decline at an early stage.

## Highlights

• We performed an epigenome-wide assessment of DNA methylation in Alzheimer's disease, mild cognitive impairment, and control whole blood.
• We observed hypermethylation of *HOXB6* in AD, which was validated via pyrosequencing.
• Network analysis (weighted gene correlation network analysis) showed differences in immune system pathways in disease.

## 1 Introduction

With an increasingly aging population the prevalence of dementia is expected to almost double in the coming 20 years, with Alzheimer's disease (AD) being the greatest contributor. AD presents itself as a heterogeneous, multifaceted disease, and this complexity is reflected in the challenges researchers face in elucidating the exact mechanisms underlying this disorder. A number of genome-wide association studies (GWAS) have identified susceptibility loci associated with the more common, sporadic form of AD (Lambert et al., 2013). However, these do not account fully for disease risk, and the exact processes involved in the development and progression of this neurodegenerative disorder remain unknown.

A growing number of studies have investigated the role of epigenetic mechanisms in the etiology and progression of AD. Epigenetic mechanisms refer to the reversible regulation of gene expression that occurs independently of the underlying DNA sequence. One such mechanism is DNA methylation, which involves the addition of a methyl group to an unmodified base, most commonly cytosine (yielding 5-methylcytosine: 5mC), and plays a critical role in the regulation of gene expression (Maunakea et al., 2013; Varley et al., 2013; Ziller et al., 2013). Recently, numerous epigenome-wide association studies (EWAS) have explored DNA methylomic variation in postmortem human brain tissue from AD patients and elderly controls and have highlighted a number of loci that show robust differences in DNA methylation in the cortex across independent cohorts (Altuna et al., 2019; De Jager et al., 2014; Gasparoni et al., 2018; Lardenoije et al., 2019; Lunnon et al., 2014; Smith et al., 2018, Smith et al., 2019, Smith et al., 2020 Watson et al., 2016). There is communication between the brain and the blood, especially in disease. In recent years, several studies have identified transcriptomic (Booij et al., 2011; Fehlbaum-Beurdeley et al., 2012; Lunnon et al., 2012, 2013, 2017; Rye et al., 2011) or proteomic (Hye et al., 2006; O'Bryant et al., 2010, 2011, 2016) alterations in the blood early in the disease and these signatures have been used for identifying novel dysfunctional pathways and biomarkers in the blood. Although valuable, the use of systemic gene expression or protein markers for this purpose still yields some pitfalls due to the dynamic nature of gene and protein expression. For example, sampling methods can significantly alter the expression levels by inducing ex vivo mRNA expression (Asare et al., 2008; Thach et al., 2003). Similarly, differences in processing methods between researchers (e.g., handling methods, sample processing methods) can affect the quality of mRNA and protein and impinge on downstream analyses (Vartanian et al., 2009; Zhao et al., 2012). DNA methylation levels are reported to be more stable than mRNA levels (Paziewska et al., 2014) and as such studying this in AD blood could be more informative of important biological pathways specifically altered in disease. To date, most blood DNA methylation studies have focused on specifically investigating candidate genes (da Silva et al., 2014; Furuya et al., 2012b,a; Wang et al., 2008). Four EWAS of AD blood have been published so far, which have identified a number of disease-associated loci. However, these studies used a limited set of (nondemented) samples and/or did not include any individuals with mild cognitive impairment (MCI) (Kobayashi et al., 2016; Lardenoije et al., 2019; Lunnon et al., 2014; Madrid et al., 2018).

To understand changes in the blood related to the development and progression of AD, it is important to include MCI individuals in addition to AD patients and controls. Often viewed as an early stage of AD, MCI is characterized by memory and other cognitive complaints and impairment, although these have no significant impact at this stage on daily living, as is seen in AD (Petersen et al., 1999). Although individuals with MCI may remain stable over time or develop another neurodegenerative disorder, MCI subjects, particularly those with amnestic MCI (aMCI), are at an increased risk of progressing to AD (Jicha et al., 2006). In these cases, the early clinical symptoms reflect the underlying pathological changes related to AD that occur years before the disease fully manifests (Hardy, 1997; Jack et al., 2010). Previous research has shown that disease-related changes in gene expression can be detected in peripheral blood from individuals with MCI and AD, with results indicating that some peripheral differences in AD can be detected in MCI subjects (Lunnon et al., 2012). Taken together, the identification of blood methylation patterns related to MCI and AD is of great interest, as it may increase our understanding of peripheral, as well as central changes that occur early in the disease.

In the current study, we have generated genome-wide DNA methylation data from well-characterized control, MCI and AD subjects with detailed demographic, clinical, neuroimaging, and transcriptomic data previously collected. We have used this dataset to identify differentially methylated loci and epigenetic differences in specific biological processes in blood, which are associated with disease status, or future progression from MCI to AD.

## 2 Methods

### 2.1 Subjects

We analyzed a subset of 284 blood samples selected for our study from the larger AddNeuroMed cohort on the basis of additional phenotypic information being available, including genomic (Furney et al., 2011b), transcriptomic (Lunnon et al., 2012, 2013), and magnetic resonance imaging (MRI) data (Furney et al., 2011a,b; Westman et al., 2011). The cross-European AddNeuroMed study is aimed at the identification of biomarkers for AD (Lovestone et al., 2007, 2009) and consists of 6 sites across Europe (Kuopio, Finland; Łódź, Poland; London, United Kingdom; Perugia, Italy; Thessaloniki, Greece; Toulouse, France). Informed consent was obtained from each participant according to the Declaration of Helsinki, and ethical approval was obtained at each site. All sites followed standardized procedures.

Within our subset, the subjects were classified into one of 3 groups according to their status at sample collection: AD (n = 86), MCI (n = 109), or elderly control (CTL; n = 89). The diagnosis of AD was made according to the National Institute of Neurological and Communicative Disorders and Stroke and the Alzheimer's Disease and Related Disorders Association (NINCDS-ADRDA) criteria (McKhann et al., 1984), and the fourth edition of the Diagnostic and Statistical Manual of Mental Disorders (DSM-IV) (del Barrio, 2004). Subjects in the MCI group were mainly recruited from memory clinics and scored 0.5 on the total Clinical Dementia Rating Scale (CDR) or 0.5 or 1 on the memory category of the CDR (Morris, 1993). All MCI individuals reported memory problems but showed no significant impairment in daily living according to Petersen's criteria of MCI (Petersen et al., 1999). Further details are provided elsewhere (Liu et al., 2011). A subset of MCI subjects progressed to AD within 1 year of the baseline measurement (MCI-AD, n = 38), while others remained stable (MCI-MCI, n = 67). A subset of 4 MCI subjects converted to AD at an unknown time after baseline collection and were excluded from any conversion analysis. Elderly CTLs were defined as showing no signs of cognitive impairment. Subjects were excluded from this study if they had any other significant psychiatric or neurological illness, were younger than 65 years of age, or were not white Caucasian. MRI data were collected for 213 individuals as described previously (Simmons et al., 2011). To obtain demographic information and medical data, semistructured interviews were carried out for all subjects. A number of neuropsychological assessments were also performed, including the mini-mental state examination (MMSE) (Folstein et al., 1975). An overview of individuals included in this study can be found in Table 1.

**Table 1:** Cohort demographics

	Control	MCI		AD
		MCI-MCI	MCI-AD	
N	89	67	42^a^	86
Gender (M/F)	34/55	34/33	16/26	30/56
Age (mean ± SD)	73.8 ± 5.3	75.1 ± 5.6	76.3 ± 5.3	76.8 ± 5.6
MMSE (mean ± SD)	29 ± 1.2	27.3 ± 1.7	26.3 ± 2.2	20.8 ± 4.5
Center (N)				
Kuopio (Finland)	22	17	16	27
Łódź (Poland)	13	5	1	8
London (United Kingdom)	22	6	3	7
Perugia (Italy)	19	19	14	23
Thessaloniki (Greece)	4	16	5	16
Toulouse (France)	9	4	3	5

Subject characteristics of the 284 samples that passed QC. Shown are sample numbers (N), sex (males [M]/females [F]), mean age (± standard deviation [SD]), mean mini-mental state examination (MMSE) ± SD, and sample numbers per source (Center). Of the 109 mild cognitive impairment (MCI) subjects, 67 remained MCI-stable (MCI-MCI) over the 1 year after sample collections, while 42 converted to AD (MCI-AD), with 38 converting within 1 year of sample collections.

a Four MCI-AD subjects were excluded from the analysis of MCI to AD conversion (but included in the ANOVA analysis of baseline diagnosis), as the exact time of conversion was not known. All individuals used in this study were white Caucasian.

### 2.2 DNA methylation analysis

DNA was extracted from the blood samples collected at baseline as described by Furney et al. (2011). The DNA was tested for degradation and purity. 500 ng DNA from each sample was sodium bisulfite–treated using the Zymo EZ-96 DNA methylation kit (Zymo Research, CA, USA) according to the manufacturer's standard protocol. Samples were assessed using the Illumina Infinium Human Methylation 450K BeadChip array (450K array; Illumina, CA, USA) using an Illumina HiScan System (Illumina, CA, USA). All samples were assigned a unique code for the purpose of the experiment and randomized with respect to sex, center, and disease status to avoid batch effects, and processed in batches of 4 BeadChips.

Raw intensity data files were imported into the R statistical environment (version 3.5.2) (R Core Team, 2018) using the *wateRmelon* (Pidsley et al., 2013) package as a methylumi object. Data quality control (QC) and preprocessing was carried out using the packages *wateRmelon* and *minfi* (Aryee et al., 2014). Initial QC checks on the data included labeling checks via sex and genetical identity, and the removal of cross-hybridizing probes, probes located on the sex chromosomes, and probes containing a single nucleotide polymorphism in the probe sequence or within 10 bp (Chen et al., 2013; Price et al., 2013). The *p-filter* function was applied, followed by the *outlyx* function within the *wateRmelon* package, with none of the 284 samples identified as outliers. Quantile normalization was then carried out using the dasen function within wateRmelon, with 401,266 probes taken forward for analysis. Blood cell type proportions were calculated using the Houseman reference–based method (Houseman et al., 2012). DNA methylation data can be found on GEO under the accession number GSE144858.

Before we identified differentially methylated positions (DMPs) associated with diagnosis, we first regressed out the effect of specific covariates that correlated with the first 3 principal components of the normalized data (Supplementary Fig. 1), with these variables being age, sex, blood cell type proportion (CD4 and CD8 T lymphocytes, natural killer cells, B cells, monocytes, granulocytes), and bisulfite conversion batch. An analysis of variance (ANOVA) was then performed on the residuals from the linear regression, to test for DNA methylation differences across all 3 groups. A post-hoc Tukey's honest significant difference (HSD) test (Tukey, 1949) was applied to the results to compare methylation levels between each of the 3 diagnostic groups at baseline (CTL, MCI, AD). For the purpose of these analyses, the MCI group included both MCI-MCI and MCI-AD as both groups were classified as MCI at baseline, which was the time point when blood sampling occurred. To identify differentially methylated regions (DMRs), which represent areas of the DNA containing multiple adjacent DMPs, we used the DMPs from both the ANOVA and the individual between group Tukey's tests and applied the *comb-p* module (Pedersen et al., 2012) in Python (version 2.7.5) (Rossum and Boer, 1991), assessing regions of 1000 base pairs, with a *p*-value threshold of *p* < 0.01. We selected only regions containing ≥2 probes, and that had a multiple testing-corrected *p* < 0.05, which was corrected using the Šidák method (Šidák, 1967). To identify DMPs and DMRs relating to the future conversion from MCI to AD we performed an analysis comparing the MCI-MCI subjects and the MCI-AD subjects, by first regressing out age, sex, blood cell type proportion, batch, and baseline MMSE score. Baseline MMSE score was included as a covariate as we observed a small, but significant difference in baseline MMSE between the MCI-MCI and MCI-AD groups. We then used a linear regression to compare the 2 groups and performed *comb-p* analysis as described above. Quantile-quantile (QQ)-plots of the *p*-values from both the ANOVA and linear regression can be found in Supplementary Fig. 2.

### 2.3 Generation of weighted gene correlation networks

In order to identify clusters, or “modules”, of highly comethylated sites in the genome, we made use of the R package for weighted gene correlation network analysis (*WGCNA*) (Langfelder and Horvath, 2008). The hypothesis underlying this method is that genes that highly co-vary, share the same underlying biological processes. Prior to creating the modules, all nonvariable probes (variance < median variance) were first removed from the normalized data, leaving 200,633 probes for analysis. Samples were then clustered based on their Euclidean distance, and clustering dendrograms were visually inspected to identify outlier samples, which were not detected. Network construction and module detection was then performed in a block-wise manner and constructed irrespective of the direction of correlation between probes (unsigned). The connection strength between 2 probes was weighted using a soft threshold value of 9 in the baseline group analysis and 8 in the comparison of MCI converters to MCI nonconverters, which emphasizes high correlations over low correlations. The soft threshold values were selected using the *pickSoftThreshold* function within the *WGCNA* package. In the resulting modules, each module is identified by an arbitrarily assigned color, and the gray module is disregarded from further analyses as it contains unassigned probes. Module eigengenes (MEs) were calculated for each module, as the first principal component across probes assigned to each module. The ME is a single value for each sample and represents the shared methylation profile of the module. Modules were generated twice: once for the baseline group analysis, which compared CTL to MCI to AD, and once for the conversion analysis using only the subset of MCI-MCI and MCI-AD samples.

### 2.4 Association of modules to traits of interest

Covariates (age, sex, blood cell type proportions, and batch number) were regressed out from the MEs, and extreme outliers (exceeding >5 standard deviations) were removed. Modules were then associated with baseline diagnosis groups and traits of interest by performing pairwise Pearson or Spearman correlations for continuous or ordinal variables, respectively. Correlations were performed using dummy variables of baseline diagnosis categories to investigate all permutations of comparisons (i.e., CTL versus MCI, CTL versus AD, and MCI versus AD), with the group not used in each comparison set to NA. Additional traits of interest included number of education years, number of *APOE ε4* alleles, MMSE score, and the following structural MRI measurements: left, right, and total entorhinal cortex volume (LEV, REV, and TEV, respectively), left, right, and total hippocampal volume (LHV, RHV, and THV, respectively), ventricular volume (VV), and whole brain volume (WBV). Similarly, regression of the same covariates (with the addition of baseline MMSE score) and outlier removal was also performed for MEs generated from the MCI-MCI and MCI-AD samples. The residuals from this regression were then used to run a linear regression, comparing nonconverters to converters.

### 2.5 Module membership and probe significance

For each of the modules showing significant (*p* < 0.05) associations with one of the 3 baseline groups, conversion to AD, or traits of interest, we calculated the module membership (MM) and probe significance (PS). MM was calculated as the Pearson correlation between the methylation value of each probe and the ME values, representing the strength of association between a probe and the module it belongs to. PS represents the strength of the correlation between a probe's methylation value and the diagnosis or trait of interest, as performed by Pearson correlations for continuous traits, and Spearman correlations for ordinal traits or diagnostic groups. We correlated and plotted MM to PS for modules of interest and focused on those that showed significant positive correlations (i.e.*, r* > 0, *p* < 0.05), which would indicate that probes more integral to the module are mainly driving the association with the trait of interest. Underlying biological processes and pathways were then examined for the modules selected, using Gene Ontology (GO) and Kyoto Encyclopedia of Genes and Genomes (KEGG) pathway enrichment analyses. For modules containing a large number of probes (>10,000), we performed these pathway analyses on the probes that were central to the module (i.e., core probes). We set this threshold at 15%, thus selecting the top 15% of probes with the highest MM. Analyses were performed using the *missMethyl* package (Phipson et al., 2015), taking into account the differing number of probes covering each gene on the array.

### 2.6 Analysis of gene expression data and association with methylation data

Normalized gene expression data from Illumina Human HT-12 v3 Expression BeadChip arrays (HT-12 arrays) was obtained from a previous study by Lunnon et al. (2012), for 237 individuals included in the current study. Expression data for all genes containing DMRs identified in this study were extracted for analysis if available. For genes nominated from the diagnostic category analysis (CTL, MCI, AD), the covariates of age, sex, and cell type proportions (estimated using Houseman's reference–based method) were regressed out of the expression data and an ANOVA and subsequent Tukey's HSD were then performed on the residuals of the regression to identify diagnostic category differences in expression levels of genes containing DMRs. For genes that contained DMRs associated with the progression to AD, only the MCI-MCI and MCI-AD samples were analyzed, with age, sex, cell type proportions, and baseline MMSE score regressed out of the expression data, with a subsequent linear regression analysis performed to assess gene expression differences between MCI-MCI and MCI-AD individuals in DMR genes.

Next, methylation values within a DMR were correlated to gene expression values of an annotated gene. Methylation values that had previously been corrected for covariates (i.e., residuals) were extracted for 450K array probes located within each DMR based on genomic location of the DMRs. Pairwise Pearson correlations were then performed between the covariate-adjusted gene expression levels and covariate-adjusted methylation values, for individual 450K probes within a DMR. We also performed correlations of gene expression and mean methylation levels from all 450K probes in the DMR. To determine whether the association between gene expression and methylation differed between CTL and individuals with MCI or AD, ANOVAs were performed on gene expression levels which included an interaction term between methylation and baseline diagnostic groups (i.e., expression ~ methylation∗group). This was performed on the probe most significantly associated with the disease for each DMR, and the mean methylation value in the DMR. Similar analyses were performed on the MCI-MCI and MCI-AD subset of individuals, for DMRs associated with progression to AD.

### 2.7 Validation of the *HOXB6* differentially methylated region using pyrosequencing

For the purpose of validating our findings, we designed a pyrosequencing assay to quantify DNA methylation at the most significant sites (cg17179862 and cg03803541) within the *HOXB6* region (chr17:46681111–46682414), which was shown to be a DMR in AD relative to CTL. Pyrosequencing assays were designed with the PyroMark Assay Design software 2.0 (Qiagen). In addition to the 2 CpG sites the assay was designed for, further 3 CpG sites that were not assessed on the 450K array were also covered. Out of the original 284 samples, 264 were used for pyrosequencing. Samples were semi-randomly selected, keeping the group sample number ratios as equal as possible, and randomly distributing samples across plates. A single amplicon of 303 base pairs was amplified using designed primers, and tested for specificity (forward primer = TTTTTGGTGAGGGGGGAGT, reverse primer = CCTACCATCCCTCCCTTATCT, sequencing primer = CTCTAACTATTACCCC). The level of DNA methylation was then quantified using the Pyromark Q24 system (Qiagen), following the standard protocol as provided by the manufacturer and the Pyro Q24 CpG 2.0.6.20 software.

Pyrosequencing data QC was performed using the Pyromark Q24 software, in addition to a visual inspection of the data and signal intensities, with all 264 samples passing QC (CTL: n = 83, MCI: n = 102, AD: n = 79). DNA methylation percentages at specific CpG sites were calculated by the software and exported to the R statistical environment. Subsequently, an ANOVA was performed for each CpG site covered by the assay, as well as the average methylation value across the region. This analysis was identical to the analysis performed on the 450K data, and the covariates of age, sex, cell type proportion, and batch were included.

## 3 Results

### 3.1 Identification of differentially methylated loci in mild cognitive impairment and Alzheimer's disease blood

The cohort characteristics are shown in Table 1. We first investigated whether any individual loci showed DNA methylation differences in either MCI or AD relative to CTL using an ANOVA model after adjusting for the covariates of age, sex, cell proportions, and batch (Supplementary Table 1). No DMPs reached the experiment-wide significance threshold that has been established for the 450K array (2.4 × 10^−7^) (Saffari et al., 2018) with the smallest ANOVA *p*-value being 5.58 × 10^−6^ for probe cg26146855, of which the closest transcription start site is located in the *TFAMP1* gene. The top 1000 most significant probes resulting from the post-hoc Tukey's HSD tests comparing CTL to MCI, MCI to AD, and CTL to AD can be found in Supplementary Tables 2, 3, and 4, respectively. In addition to comparing methylation levels at baseline between the 3 groups, we were also interested in identifying differences within the MCI population that were predictive of later progression to AD. For this purpose, we compared the MCI-MCI group to the MCI-AD group. While no DMPs passed the experiment-wide significance threshold, the most significant DMP was located in the *TRIM62* gene and showed hypomethylation in converters (probe cg25342005, *p* = 1.67 × 10^−6^; Supplementary Table 5).

### 3.2 A number of significant differentially methylated regions can be identified in mild cognitive impairment and Alzheimer's disease blood

**Fig. 1:**
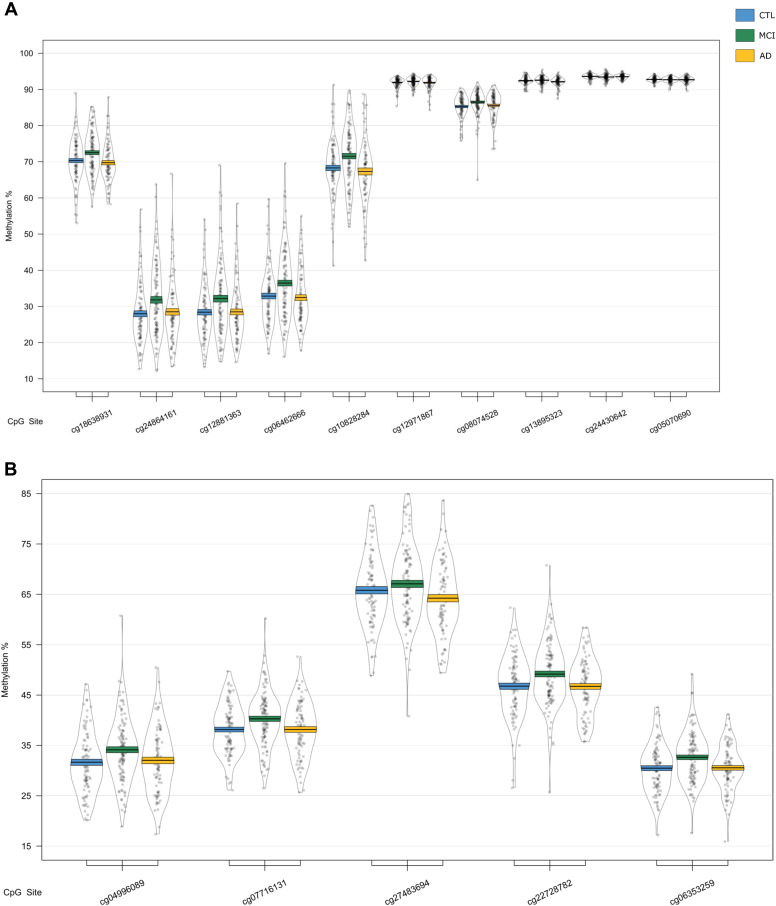

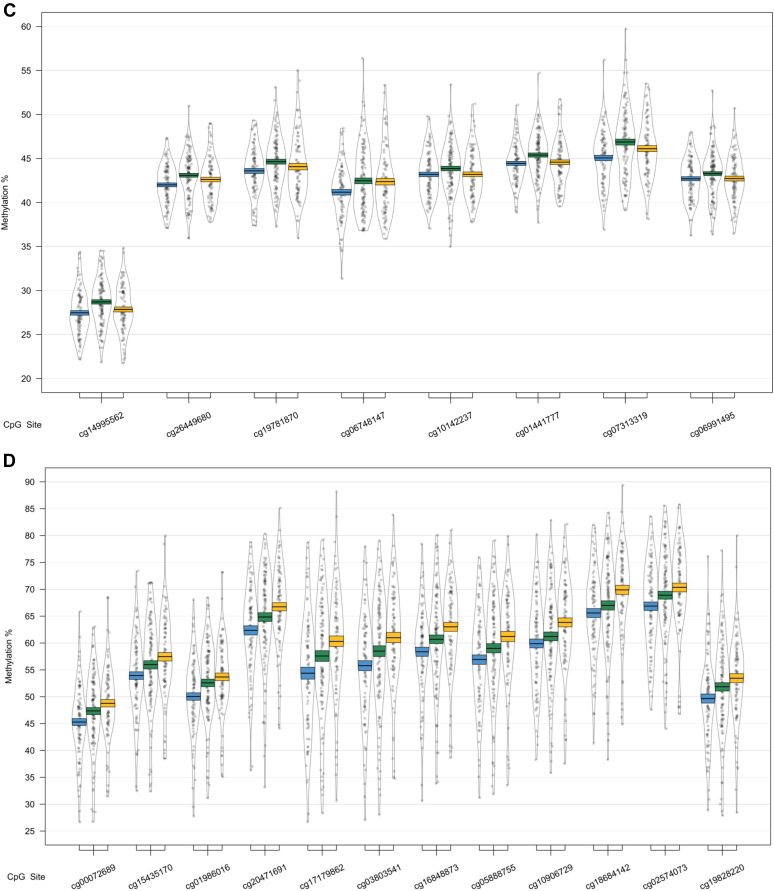
Differentially methylated regions (DMRs) in a comparison of baseline diagnosis of mild cognitive impairment and Alzheimer's disease relative to controls. DMRs shown are located in or near the genes *MOV10L1* (A), *CBFA2T3* (B), *TPTEP2-CSNK1E* (C), and *HOXB6* (D). Displayed for each DMR are the methylation levels of individual probes located within the DMR, ordered by genomic location. Methylation values have been corrected for covariates age, sex, cell type proportion, and batch.

We next used a sliding window approach to identify the regions spanning multiple adjacent DMPs that were significantly different in MCI and AD. We found 4 DMRs associated with differences across the 3 baseline groups (CTL, MCI, and AD) (Table 2A). A 10-probe DMR of 574 bp was identified in *MOV10L1* (Fig. 1A), as well as a 5-probe (582 bp) intergenic DMR annotated to *CBFA2T3* (Fig. 1B), with probes in both DMRs generally showing hypermethylation in MCI samples, with levels in AD samples similar to CTL. An 8-probe DMR of 301 bp was found in the readthrough transcription region of *TPTEP2-CSNK1E*, which appeared to be mainly driven by hypermethylation in the MCI group (Fig. 1C).

**Table 2:** Differentially methylated regions in blood

Gene	Position	Gene feature	*n*	*p*-value	Šidák-*P*	Average methylation %		
						CTL	MCI	AD
A. ANOVA: CTL versus MCI versus AD								
HOXB-AS3; HOXB6	chr 17: 46681111 - 46682414	nc_intron+nc_exon; TSS+intron+exon+utr5	12	2.79E-14	8.58E-12	56.59	58.79	60.81
MOV10L1	chr 22: 50528179 - 50528753	TSS+intron+utr5+cds; TSS+exon+utr5	10	2.03E-07	1.42E-04	68.38	70.18	68.23
CBFA2T3	chr 16: 88937216 - 88937798	Intergenic	5	2.61E-07	1.80E-04	42.57	44.66	42.34
TPTEP2-CSNK1E	chr 22: 38714166 - 38714467	intron+utr5	8	1.87E-06	2.49E-03	41.21	42.29	41.69
B. CTL vs. AD								
HOXB-AS3; HOXB6	chr 17: 46681111 - 46682414	nc_intron+nc_exon; TSS+intron+exon+utr5	12	3.36E-16	1.03E-13	56.59	58.79	60.81
Gene	Position	Gene feature	*n*	*p*-value	Šidák-*P*	Average methylation %		
						MCI-MCI	MCI-AD	
C. MCI-MCI vs. MCI-AD								
CHKB-CPT1B; CPT1B; CHKB	chr 22: 51016501 - 51017433	nc_intron; TSS+intron+exon+utr5; exon+utr3	14	2.05E-14	8.84E-12	64.92	61.26	
SMC1B; RIBC2	chr 22: 45809319 - 45810044	TSS+intron+utr5+cds; TSS+intron+utr5+cds	15	8.26E-09	4.57E-06	24.62	26.62	
TMEM184A	chr 7: 1595602 - 1596261	TSS+intron+exon+utr5	6	2.41E-08	1.47E-05	45.33	43.31	
KCNAB3	chr 17: 7832680 - 7833238	TSS+cds	7	8.11E-08	5.83E-05	80.31	76.73	
GABBR1	chr 6: 29599012 - 29599391	intron+exon+utr5; intron+cds	10	9.72E-08	1.03E-04	63.80	61.48	
FIGN	chr 2: 164204628 - 164205344	Intergenic	6	3.58E-07	2.01E-04	52.84	56.10	
PRDM1	chr 6: 106546704 - 106546825	TSS+exon+utr5; intron	5	1.04E-07	3.45E-04	62.01	58.89	
FLJ37453	chr 1: 16163555 - 16164123	nc_intron	6	5.92E-07	4.18E-04	29.67	27.34	
OR56A3; TRIM5	chr 11: 5959658 - 5960214	Intergenic	5	9.68E-07	6.98E-04	81.17	77.54	

Differentially methylated regions (DMRs) in a comparison of control (CTL), mild cognitive impairment (MCI), and Alzheimer's disease (AD) blood samples. Shown are DMRs for (A) the overall three group (ANOVA) comparison, the post-hoc (B) CTL versus AD comparison, and (C) the MCI-stable (MCI-MCI) versus MCI-converter (MCI-AD) comparison. Displayed for each region is the UCSC gene name, chromosomal position (genome build 37), gene feature (TSS = transcription start site; utr5 = 5′ untranslated region; utr3 = 3′ untranslated region; cds = coding sequence), number of probes in region (n), *p*-value and multiple testing-corrected *p*-value (Šidák-P), and average beta per group.

**Fig. 2:**
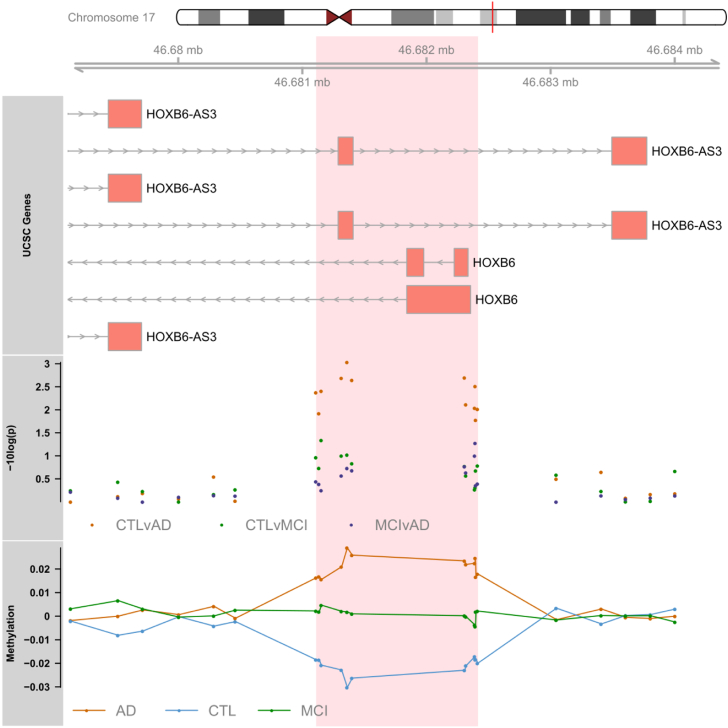
The *HOXB6* DMRs, shown to be altered in Alzheimer's disease (AD) relative to controls (CTL). The region spanned by the DMRs is highlighted in red, and genomic location and UCSC gene annotations are shown, in addition to a mini-Manhattan plot of the *p*-values of probes within and neighboring the DMR, *p*-values related to changes in AD relative to CTL are shown in orange, mild cognitive impairment (MCI) relative to CTL in green, and AD relative to MCI in purple. The bottom panel shows relative methylation levels across the region, with methylation in AD in orange, MCI in green, and CTL in blue. (For interpretation of the references to color in this figure legend, the reader is referred to the Web version of this article.)

One of the 4 identified DMRs was driven by a difference between the CTL and AD groups (Table 2B); we identified a 1303 bp DMR in the *HOXB6* gene, containing 12 probes (Fig. 1, Fig. 2). Each of the 12 probes showed hypermethylation in AD.

**Fig. 3:**
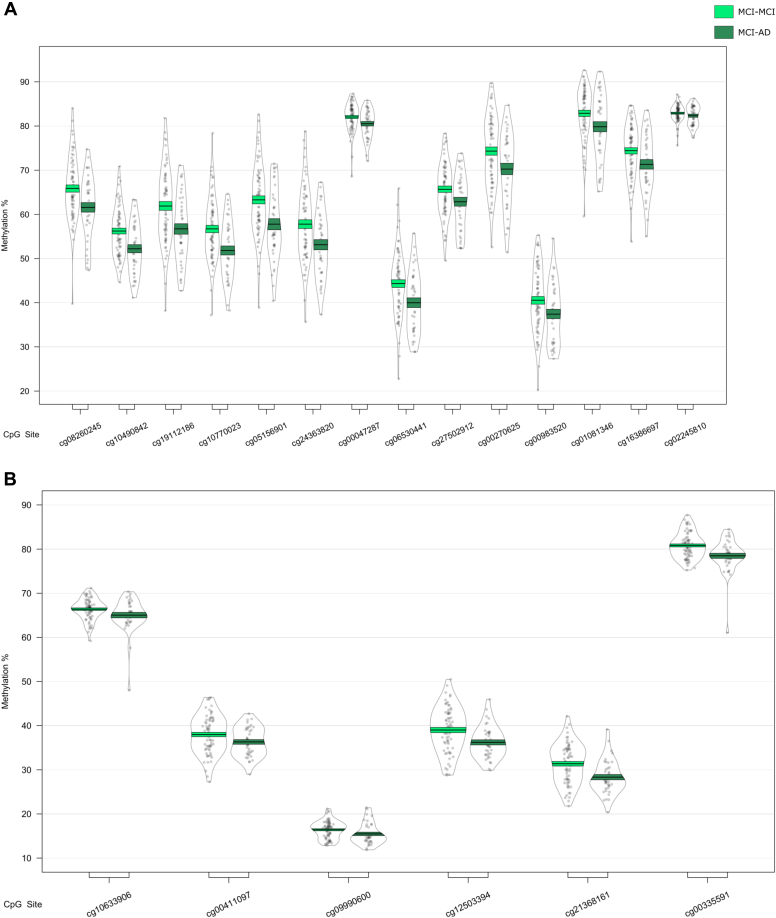

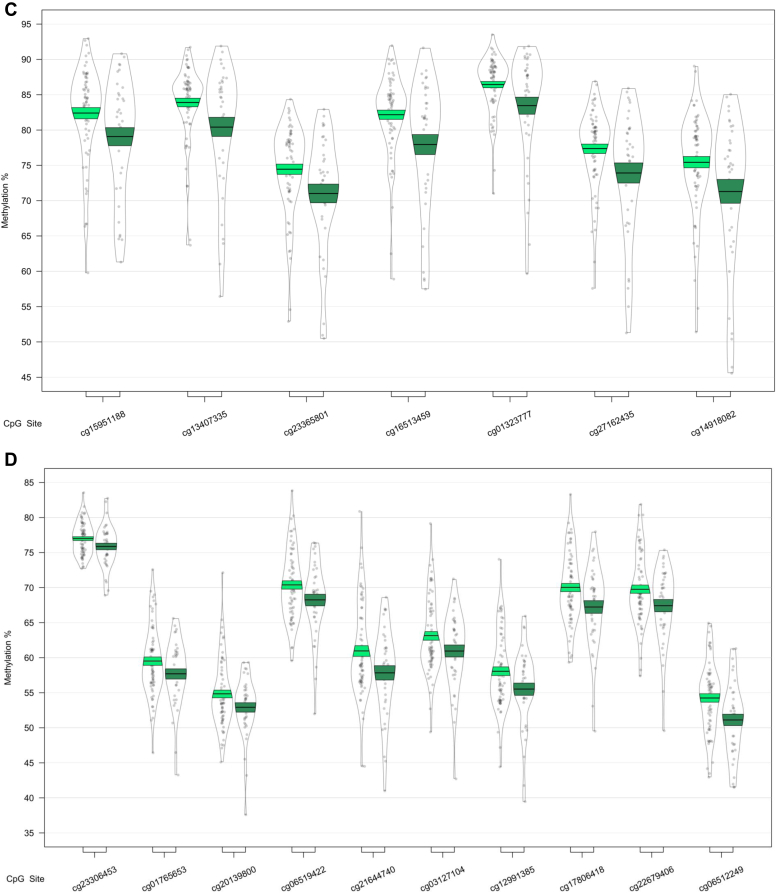

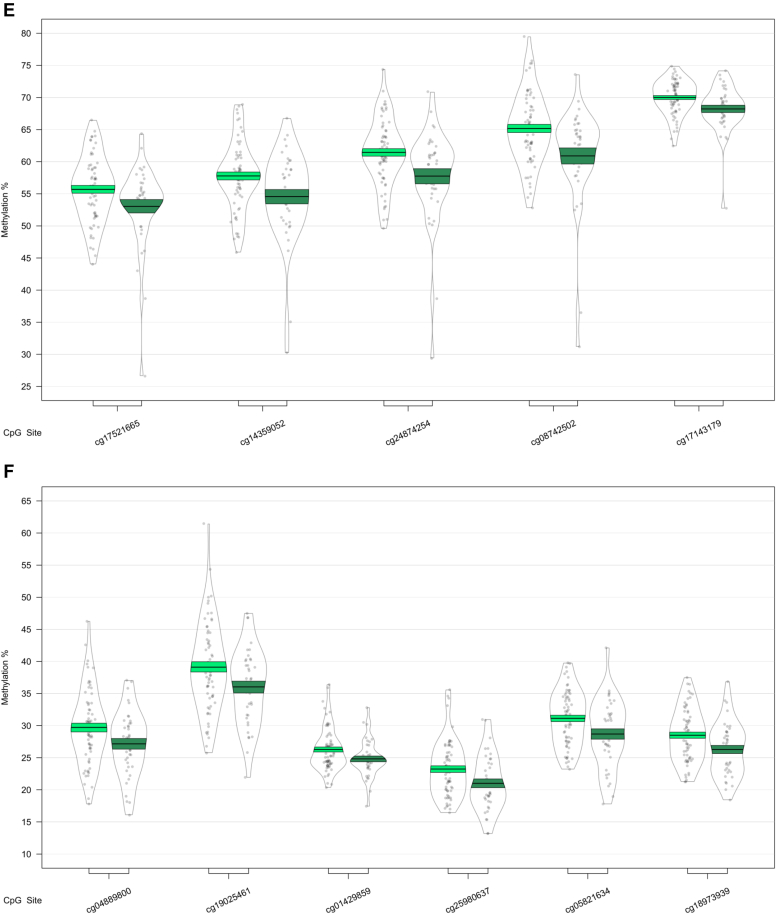

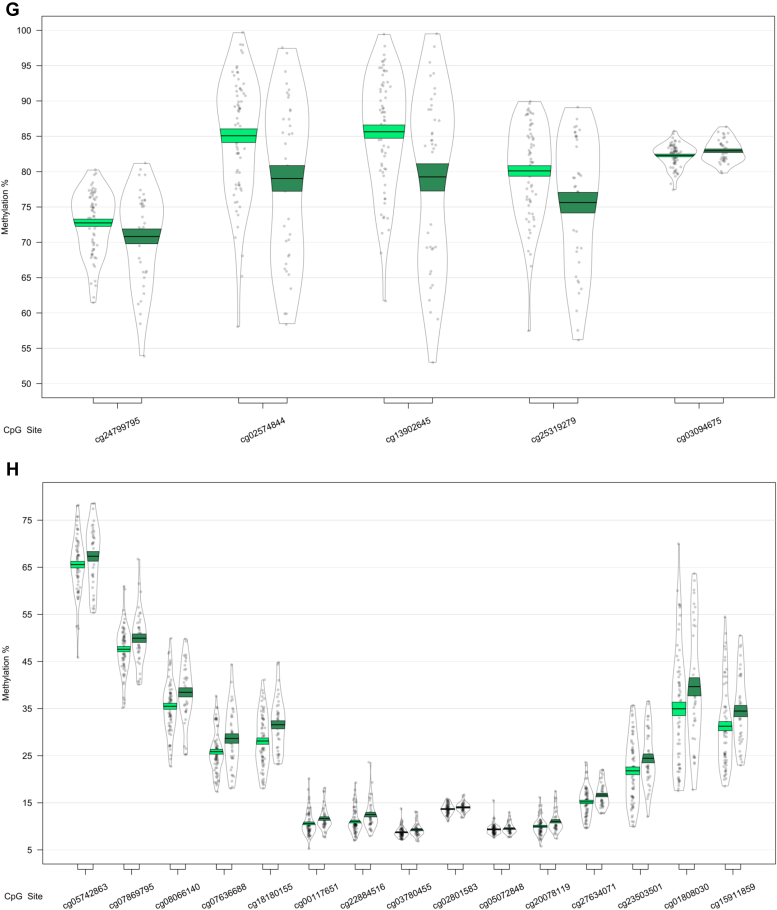

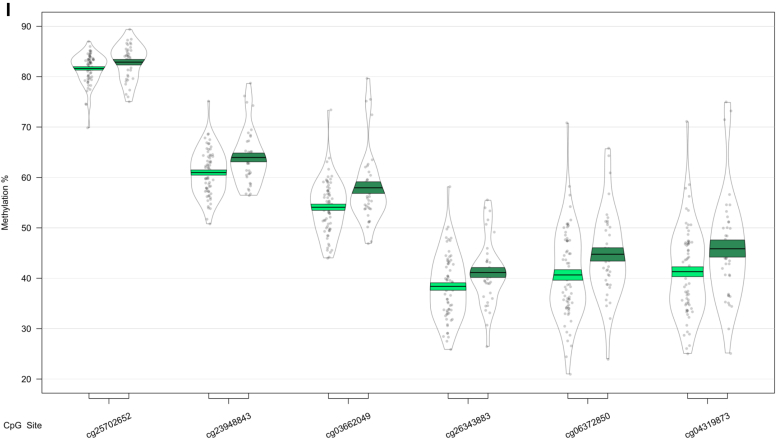
Differentially methylated regions (DMRs) in a comparison of mild cognitive impairment (MCI) individuals who converted to Alzheimer's disease (AD) within 1 year after baseline assessment (MCI-AD; dark green, shown on the right) and those who remained stable (MCI-MCI; light green, shown on the left). DMRs shown are located in or near the genes *CPT1B* (A), *TMEM184 A* (B), *KCNAB3* (C), *GABBR1* (D), *PRDM1* (E), *FLJ37453* (F), *OR56A3* and *TRIM5* (G), *SMC1B* and *RIBC2* (H), and *FIGN* (I). Displayed for each DMR are the methylation levels of all probes (*p* < 0.05) within the genomic location covered by each DMR, ordered by genomic location. Methylation values have been corrected for covariates age, sex, cell type proportion, batch, and baseline mini-mental state examination score. (For interpretation of the references to color in this figure legend, the reader is referred to the Web version of this article.)

In our analysis of MCI conversion to AD, we identified 9 significant DMRs (Table 2C; Fig. 3). We found DMRs showing decreased methylation in MCI-AD converters relative to MCI-MCI nonconverters in the genes *CPT1B* and *CHKB* (932 bp; 14 probes) (Fig. 3A), *TMEM184 A* (659 bp; 6 probes) (Fig. 3B), *KCNAB3* (558 bp; 7 probes) (Fig. 3C), *GABBR1* (379 bp; 10 probes) (Fig. 3D), *PRDM1* (121 bp; 5 probes) (Fig. 3E), *FLJ37453* (568 bp; 6 probes) (Fig. 3F), and *OR56A3* and *TRIM5* (556 bp; 5 probes) (Fig. 3G). Hypermethylation in MCI-AD converters relative to MCI-MCI nonconverters was seen in 2 DMRs located in the genes *SMC1B* and *RIBC2* (725 bp; 15 probes) (Fig. 3H), and an intergenic region near the gene *FIGN* (716 bp; 6 probes) (Fig. 3I).

### 3.3 Validation of the Alzheimer's disease–associated differentially methylated region in HOXB6 by pyrosequencing

**Fig. 4:**
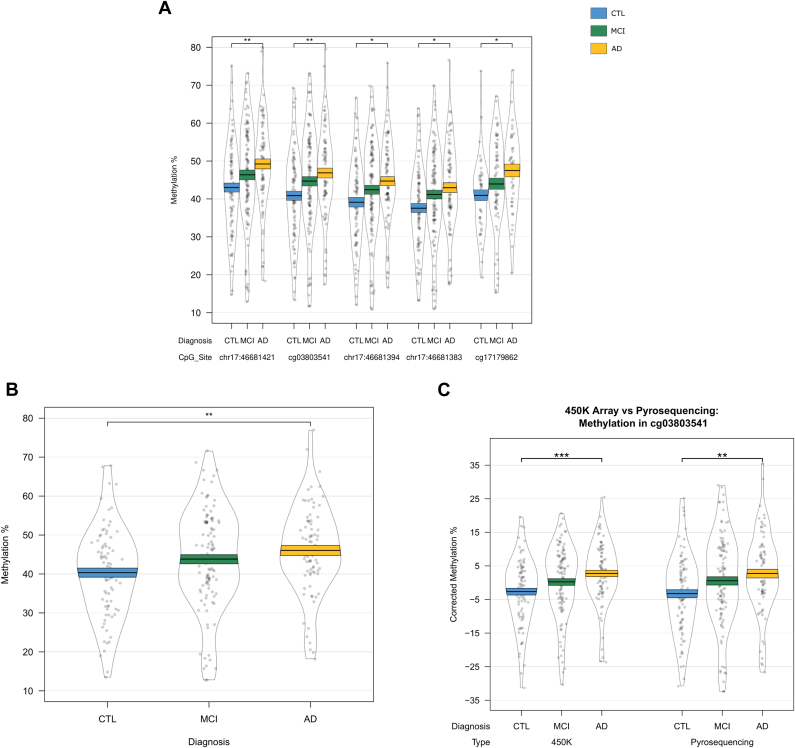

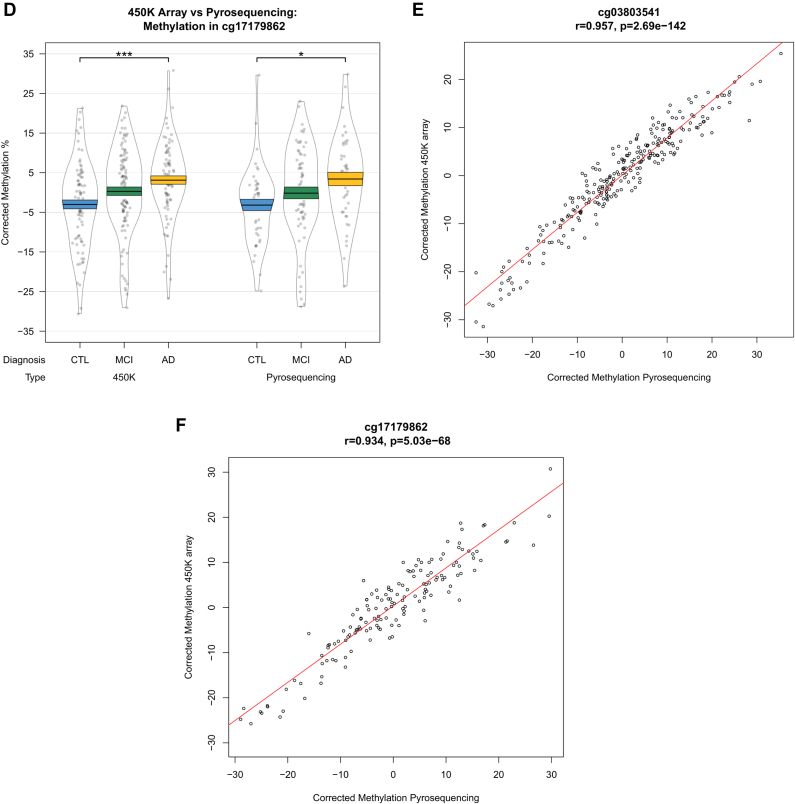
Validation of the HOXB6 differentially methylated region. DNA methylation was assessed via pyrosequencing and significant changes were found between controls and individuals with Alzheimer's disease, but not mild cognitive impairment at all 5 CpG sites assessed (A) and when averaged over all 5 probes (B). DNA methylation patterns quantified by the Illumina 450K array and pyrosequencing were similar for cg03803541 (C) and cg17179862 (D) and were significantly correlated (E and F, respectively).

Interestingly, differential DNA methylation at the most significant locus within the *HOXB6* DMR (cg17179862) has been previously reported in AD hippocampus (Altuna et al., 2019). To further explore AD-associated hypermethylation in this gene, we used pyrosequencing to validate our *HOXB6* DMR, covering 2 CpG sites on the array (cg17179862, cg03803541) as well as 3 neighboring CpG sites that were not covered by the 450K array (chr17:46681421, chr17:46681394, and chr17:46681383). We found significant differences between groups at all 5 CpG sites (Supplementary Table 6, Fig. 4A), and when averaged over the full 5 probes (Fig. 4B), demonstrating hypermethylation in AD samples relative to controls. The pattern of DNA methylation quantified by the 450K array and pyrosequencing was similar for both cg03803541 (Fig. 4C) and cg17179862 (Fig. 4D), with a significant correlation of the methylation values estimated by the 2 technologies for both cg03803541 (Fig. 4E: r = 0.957, *p* = 2.69 × 10^−142^) and cg17179862 (Fig. 4F: r = 0.934, *p* = 5.03 × 10^−68^).

### 3.4 Transcriptional differences in genes containing differentially methylated regions

To explore the relationship between DNA methylation and expression, we first assessed whether the expression levels of genes containing the 4 baseline diagnosis-associated DMRs or the 9 conversion DMRs were different in the diagnostic groups. Expression data were only available for 2 of the 6 genes we identified as containing DMRs in the baseline group analysis (*HOXB6,* and *CSNK1E* associated with the readthrough transcription region of *TPTEP2-CSNK1E*) (Supplementary Table 7). Of these 2 genes, *CSNK1E,* which had shown increased DNA methylation in MCI samples, also showed a significant difference in gene expression between groups (F = 15.94, *p* = 3.25 × 10^−7^). More specifically, we observed significantly increased mRNA expression in both MCI and AD subjects relative to control (Tukey's *p* = 1.46 × 10^−7^ and *p* = 0.003, respectively, Supplementary Fig. 3A). Although there was significantly higher gene expression and DNA methylation (across the DMR), there was no correlation of expression and methylation across all samples, or when we performed correlations separately in the 3 diagnostic groups (Supplementary Fig. 3C, Supplementary Table 8). Although we did not observe any significant differences in gene expression for *HOXB6* (Supplementary Fig. 4A), we did find a correlation of expression and methylation when performing correlations in the AD group only (*r* = -0.24, *p* = 0.041) (Supplementary Fig. 4C, Supplementary Table 8).

Expression data were also available for 5 of the 9 significant DMRs we identified in our analysis of progression from MCI to AD (*GABBR1, PRDM1, FLJ37453, TRIM5,* and *CPT1B/CHKB*). The *CPT1B/CHKB* DMR was covered by 3 probes on the gene expression microarray, one probe measuring *CPT1B* expression and 2 probes measuring *CHKB* expression (ILMN_2331205 and ILMN_1659054). Although none of these genes showed differential expression in MCI subjects who progressed to AD (Supplementary Table 9), *CPT1B/CHKB* showed a significant positive correlation of methylation across the DMR and *CPT1B* gene expression (Supplementary Table 10). The average methylation level across the *CPT1B/CHKB* DMR was significantly correlated with gene expression across all samples (*r* = 0.40, *p* = 8.62 × 10^−5^, Supplementary Fig. 5), which appeared to be primarily driven by a correlation observed in the MCI-MCI samples (*r* = 0.49, *p* = 7.27 × 10^−5^) and not the MCI-AD samples.

### 3.5 Clusters of methylated loci associated with mild cognitive impairment and Alzheimer's disease

**Fig. 5:**
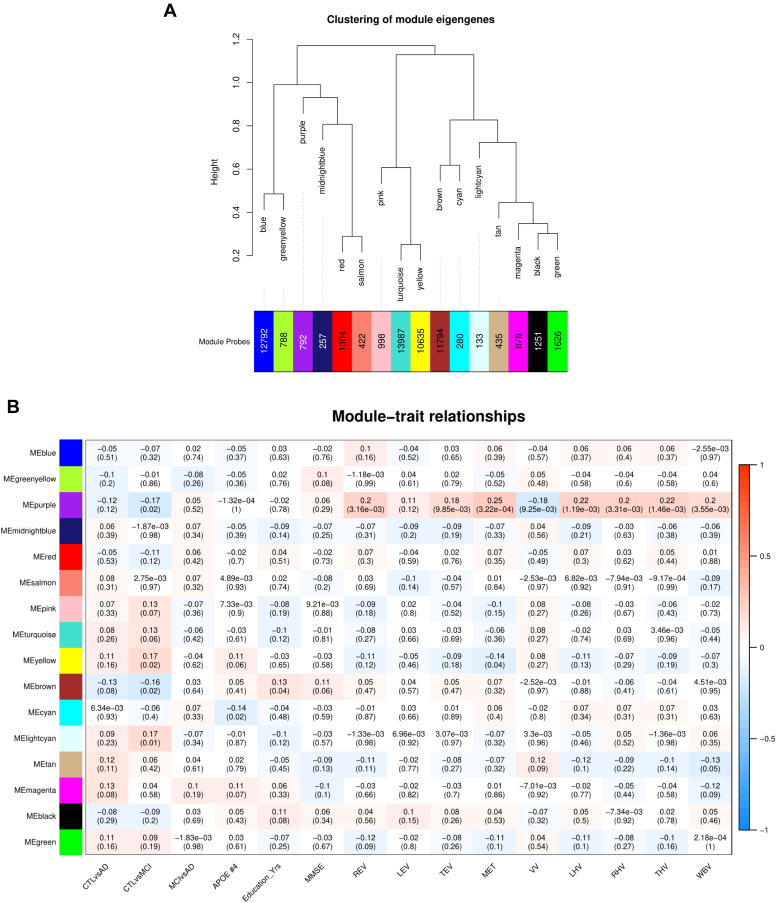
Clusters (or “modules”) of highly comethylated loci identified in the full dataset of 284 samples (A). Modules are hierarchically clustered based on calculated module eigengenes (representative of the methylation values within each module), and the number of probes included in each module are indicated along the x-axes. The color of each module is assigned in an arbitrary manner. (B) Correlations between module eigengenes and traits of interest, with module names shown along the y-axis. Correlation estimates are reported, with *p*-values in parentheses. Spearman correlations were performed for the controls (CTL) versus Alzheimer's disease (AD) comparison, CTL versus mild cognitive impairment (MCI) comparison, the MCI versus AD comparison, and the number of *APOE*-ε4 alleles (APOE #4). Pearson correlations were calculated for the number of education years (Education_Yrs); mini mental state examination (MMSE) scores; and the following structural imaging measurements: right, left, and total entorhinal volume (REV, LEV, and TEV, respectively); mean entorhinal thickness (MET); ventricular volume (VV); left, right and total hippocampal volume (LHV, RHV, and THV, respectively); and whole brain volume (WBV). Modules with a correlation *p*-value <0.05 were selected for further analysis. (For interpretation of the references to color in this figure legend, the reader is referred to the Web version of this article.)

To identify clusters of probes that are comethylated and are therefore hypothesized to share a common function, we performed WGCNA and classified the entire filtered data set of 200,633 probes into 16 modules (Fig. 5A). These modules were correlated to the group comparisons of diagnostic status at baseline, as well as to several other traits of interest (Fig. 5B, Supplementary Table 11), after controlling for covariates. The brown module, which consists of 11,794 probes, was negatively correlated with differences between CTL and MCI (*ρ* = −0.16, *p* = 2.31 × 10^−2^) and correlated positively with an individual's number of education years (*r* = 0.13, *p* = 3.59 × 10^−2^). Three more modules also showed a correlation with MCI versus CTL; the light cyan module consisting of 133 probes (*ρ* = 0.18, *p* = 1.2 × 10^−2^), and the yellow module which consists of 10,635 probes (*ρ* = 0.17, *p* = 1.51 × 10^−2^). The yellow module further correlates to the structural imaging variable MET (*r* = −0.14, *p* = 4.26 × 10^−2^). The purple module (792 probes) also correlates to MCI versus CTL (*ρ* = −0.17, *p* = 1.98 × 10^−2^), as well as the majority of structural imaging variables: REV (*r* = 0.21, *p* = 3.16 × 10^−3^), TEV (*r* = 0.18, *p* = 9.85 × 10^−3^), MET (*r* = 0.25, *p* = 3.22 × 10^−4^), VV (*r* = -0.18, *p* = 9.25 × 10^−3^), LHV (*r* = 0.22, *p* = 1.19 × 10^−3^), RHV (*r* = 0.20, *p* = 3.31 × 10^−3^), THV (*r* = 0.22, *p* = 1.46 × 10^−3^), and WBV (*r* = 0.20, *p* = 3.55 × 10^−3^). Finally, the cyan module (280 probes) correlates to an individual's number of *APOE ε4* alleles (*ρ* = −0.14, *p* = 1.75 × 10^−2^).

Subsequently, we investigated whether the probes that are integral to a specific module are also the probes that are driving the association with the relevant diagnosis or trait. We did this by correlating and plotting MM and PS values, and focusing on those modules that showed positive (*r* > 0, *p* < 0.05) correlations between MM and PS (Supplementary Table 12). Significant positive MM to PS correlations were found in the brown (*r* = 0.26, *p* = 5.93 × 10^−179^), purple (*r* = 0.19, *p* = 9.18 × 10^−8^), and yellow (*r* = 0.25, *p* = 6.64 × 10^−153^) modules in association with CTL versus MCI. The brown module further showed significant positive MM to PS correlations in relation to education years (*r* = 0.11, *p* = 2.40 × 10^−32^). The yellow module displayed a positive MM to PS correlation (*r* = 0.22, *p* = 6.42 × 10^−117^) in association with MET, and the cyan module showed a positive MM to PS correlation in association with the number of *APOE* ε4 alleles (*r* = 0.20, *p* = 6.42 × 10^−4^). These modules were the primary focus of our pathway analyses. MM and PS plots for these modules are shown in Supplementary Fig. 6; for a full overview of all MM and PS correlations, see Supplementary Table 12.

### 3.6 Functional role of modules associated with mild cognitive impairment and Alzheimer's disease

We sought to identify the pathways that were enriched in modules that were affected in disease or were associated with certain traits. For this purpose, we performed GO and KEGG enrichment analyses, with for large (i.e., yellow and brown) modules only the core probes being used for the enrichment analyses. Pathways related to the brown (Supplementary Fig. 7), purple, yellow (Supplementary Fig. 8), and cyan (Supplementary Fig. 9) modules all passed false discovery rate (FDR) multiple testing correction. A large number of GO terms were associated with the core of the brown module, which was related to MCI relative to CTL as well as number of education years, among which were “extracellular matrix” (*q* = 4.23 × 10^−7^), “channel activity” (*q* = 3.19 × 10^−5^), and “passive transmembrane transporter activity” (*q* = 3.19 × 10^−5^) (Supplementary Fig. 7A). Furthermore, KEGG terms related to this module included “Protein digestion and absorption” (*q* = 1.06 × 10^−2^), “Oxytocin signaling pathway” (*q* = 1.06 × 10^−2^), and “Regulation of actin cytoskeleton” (*q* = 1.10 × 10^−2^) (Supplementary Fig. 7B). The core of the yellow module showed differences related to MCI, relative to CTL, as well as MET, and we found in our enrichment analyses of the core probes that the top GO terms included “leukocyte activation” (*q* = 7.46 × 10^−13^), “cell activation” (*q* = 7.46 × 10^−13^), and “immune response” (*q* = 5.84 × 10^−11^), while the top KEGG terms included “platelet activation” (*q* = 1.93 × 10^−2^), “adrenergic signaling in cardiomyocytes” (*q* = 1.93 × 10^−2^), and “sphingolipid signaling pathway” (*q* = 2.34 × 10^−2^) (Supplementary Fig. 8). The purple module, which was also associated with differences related to MCI relative to CTL, was connected with one GO term; “vesicle-mediated transport” (*q* = 4.35 × 10^−2^), but no significant KEGG terms. Finally, the cyan module, which was associated with the number of *APOE ε4* alleles, was related to a number of GO terms, including “cell activation” (*q* = 3.07 × 10^−4^), “regulation of cell adhesion” (*q* = 4.51 × 10^−4^), “leukocyte activation” (*q* = 4.51 × 10^−4^), and “regulation of cell death” (*q* = 8.32 × 10^−4^) (Supplementary Fig. 9) and one KEGG pathway: “T cell receptor signaling pathway” (*q* = 3.73 × 10^−2^).

### 3.7 Investigating clusters of comethylated loci associated with progression to Alzheimer's disease

In addition to modules associated with baseline diagnosis of MCI and AD, we also identified 31 modules of highly comethylated loci in the subset of MCI-MCI and MCI-AD samples (Supplementary Fig. 10). Only one of these modules, the orange module, was shown to be significantly associated with future progression to AD (*β* = -0.04, *p* = 4.38 × 10^−2^; Supplementary Table 13). We then correlated the MM to the PS for this module and found a significant positive correlation (*r* = 0.36, *p* = 9.40 × 10^−6^). Following GO and KEGG pathway analysis, we found no GO terms passing FDR multiple testing correction, but top KEGG terms included “renal cell carcinoma” (*q* = 1.21 × 10^−2^), “nonhomologous end-joining” (*q* = 2.00 × 10^−2^), and “ErbB signaling pathway” (*q* = 2.00 × 10^−2^) (Supplementary Fig. 11).

## 4 Discussion

The present study, which reflects the first large-scale EWAS of AD blood samples, identified epigenetic signatures related to AD and MCI, as well as signatures associated with future conversion from MCI to AD.

The *HOXB6* gene contained a DMR that reflected differences in methylation in AD relative to CTL, which were validated using pyrosequencing. *HOXB6* encodes the homeobox protein B6, which is part of a larger cluster of homeobox B genes located on chromosome 17. Homeobox genes are DNA-binding proteins that have been implicated in early body morphogenesis (Krumlauf, 1994) as well as hematopoietic development. Specifically, *HOXB6* has been shown to be required for normal generation of granulocytes and monocytes (Giampaolo et al., 2002). Interestingly, a recent EWAS of AD hippocampus has shown DNA methylation differences in cg17179862, which was the most significant probe in the DMR we identified and validated (Altuna et al., 2019). The study by Altuna et al. further showed that increased methylation at this locus was positively correlated with tau burden.

*MOV10L1,* which was associated with differences between all 3 groups, encodes an RNA helicase. This protein was shown to be crucial for the production of Piwi-interacting RNAs (piRNAs) by Vourekas et al. (2015). PiRNAs represent small noncoding RNAs involved in epigenetic regulation, which can bind to PIWI proteins and may induce gene silencing via DNA methylation (Aravin et al., 2008; Girard et al., 2006), or RNA-cleavage (for a review, see Luteijn and Ketting (2013)). Although initially believed to be mainly present in germline cells, piRNAs have been shown to be stably expressed in human blood (Yang et al., 2015) and have also been shown to be downregulated in tumor tissue and upregulated in blood of renal carcinomas (Iliev et al., 2016). Interestingly, Watson et al. (2016) performed an EWAS of AD superior temporal gyrus and identified a DMR spanning 13 probes, including all ten probes we identified in the *MOV10L1* gene in the current study. Of note, where Watson et al. detected AD-related hypermethylation in these 10 probes, we found hypermethylation in MCI when compared with AD and CTL individuals, while methylation levels of AD subjects were not distinct from CTL individuals.

Of the 9 DMRs that were related to future conversion to AD, our most significant region was located in *CPT1B*, which encodes the protein carnitine palmitoyltransferase 1B. Differential DNA methylation in *CPT1B* has been previously identified in blood and fetal cortex of patients with Down syndrome (El Hajj et al., 2016; Kerkel et al., 2010). This is interesting as individuals with Down syndrome often develop AD as a result of trisomy of chromosome 21, causing them to have an additional copy of the amyloid precursor protein (*APP*) gene. The study by El Hajj et al. (2016) identified a DMR in *CPT1B* consisting of 18 probes in Down syndrome fetal cortex samples, which spanned the region discovered in the present study. They detected hypermethylation in 13 probes in Down syndrome, while we observed hypomethylation in those MCI individuals who convert to AD. Kerkel et al. (2010) similarly detected hypermethylation at one CpG site in our *CPT1B* DMR in peripheral blood leukocytes of individuals with Down syndrome, concomitant with significant overexpression of the gene. While we observed hypomethylation of the DMR, the positive relationship found between methylation and expression for this region was validated in our study. Of note, overexpression of *CPT1B* has also been found in blood from soldiers with post-traumatic stress disorder (Zhang et al., 2015), a known risk factor for developing AD (Agís-Balboa et al., 2017; Yaffe et al., 2010). Interestingly, no overlap was found between DMRs associated with conversion and DMRs related to diagnosis at baseline. This may reflect limited power in our MCI conversion analysis due to sample size or could reflect temporal patterns of DNA methylation in the process of conversion from MCI to AD.

In addition to DMRs, by using WGCNA and subsequent pathway analyses we further identified biological mechanisms affected in disease. The cyan module that was linked to the number of *APOE* ε4 alleles is involved in GO pathways related to the immune system, which is interesting given that the immune system is known to be activated in AD (Heppner et al., 2015), and as *APOE* ε4 is the strongest genetic risk factor for sporadic AD (Lambert et al., 2013). The core of the brown module, which reflects methylomic differences related to an individual's number of education years and differences in MCI relative to CTL, was shown to be involved in transmembrane processes (GO), as well as oxytocin signaling (KEGG). The oxytocin signaling pathway is linked to social behaviors, as well as several psychiatric disorders (e.g., depression) (Feldman et al., 2016) Interestingly, a DMR was recently identified in the oxytocin gene (*OXT*), which was hypomethylated in AD brain (Lardenoije et al., 2019; Watson et al., 2016) and hypermethylated in the blood in individuals who subsequently converted to AD (Lardenoije et al., 2019). Oxytocin is involved in the modulation of stress, social behaviors, and associative learning (Olff et al., 2013), and altered levels of oxytocin have been reported in AD postmortem brain tissue (Mazurek et al., 1987) and cerebrospinal fluid (North et al., 1992). It is interesting that the sphingolipid signaling pathway is found in the KEGG results from the core probes of the yellow module related to differences in MCI relative to CTL. Multiple studies have indicated that sphingolipid signaling pathways are implicated in AD (Crivelli et al., 2020), and the measurement of lipids in the pathway is being explored as a potential biomarker of AD and neurodegeneration (Mielke and Lyketsos, 2010). Similar to the cyan module, the majority of GO terms in the core of the yellow module are related to various processes of immune activation. In our network analysis examining conversion from MCI to AD, we identified a pathway in the orange module associated with nonhomologous end-joining. Nonhomologous end-joining activity is involved in repairing the double-strand DNA breaks and has been reported to be decreased in AD brain (Kanungo, 2013; Shackelford, 2006).

In summary, this is the first EWAS to identify epigenetic signatures and functional pathways specific to MCI, AD, and conversion to AD in the blood. However, there are some limitations to our study. First, we have profiled DNA methylation patterns in whole blood, and it is known that there are subtle alterations in the abundance of specific blood cell types in MCI and AD (Lunnon et al., 2012). Although we have controlled for the proportions of these different cells, it will be of interest to investigate disease-associated signatures in individual cell types. Second, individuals were only followed up clinically for up to 2 years following the baseline assessment and further studies should profile cohorts consisting of CTL and MCI subjects with long-term clinical follow-up to identify the preclinical changes. In addition, biomarkers were not available to support the clinical diagnosis of AD. Third, our comparisons of DNA methylation and gene expression were limited to only those genes with variable expression levels in the previous study (Lunnon et al., 2012) and did not examine transcript variants. Fourth, we have not replicated our findings in an independent study cohort. Although we did validate our *HOXB6* DMR in the same samples using an alternative technology, in the future it will be interesting to verify the loci we identified in a different set of samples. Finally, although there is some communication between the brain and the blood, not all differences found to be associated with AD in the blood may be functionally related to the processes taking place in the brain. Differences in DNA methylation may be the result of parallel effects or comorbidities, and may not be causally related to disease, but could reflect mediating or downstream effects. It would be interesting for future studies to explore the exact role of the epigenetic signatures identified in this study, and to explore their potential as biomarkers for an early diagnosis of AD and therapeutic targets.

## CRediT authorship contribution statement

**Janou A.Y. Roubroeks:** Conceptualization, Methodology, Software, Writing - original draft, Writing - review & editing, Formal analysis, Investigation. **Adam R. Smith:** Investigation, Validation, Writing - review & editing. **Rebecca G. Smith:** Methodology, Writing - review & editing. **Ehsan Pishva:** Methodology, Formal analysis, Writing - review & editing. **Zina Ibrahim:** Formal analysis, Resources. **Martina Sattlecker:** Formal analysis, Writing - review & editing, Resources. **Eilis J. Hannon:** Methodology, Writing - review & editing. **Iwona Kłoszewska:** Resources. **Patrizia Mecocci:** Resources. **Hilkka Soininen:** Resources. **Magda Tsolaki:** Writing - review & editing, Resources. **Bruno Vellas:** Resources. **Lars-Olof Wahlund:** Writing - review & editing, Resources. **Dag Aarsland:** Writing - review & editing, Resources. **Petroula Proitsi:** Resources. **Angela Hodges:** Resources. **Simon Lovestone:** Writing - review & editing, Resources. **Stephen J. Newhouse:** Resources. **Richard J.B. Dobson:** Writing - review & editing, Resources. **Jonathan Mill:** Funding acquisition, Writing - review & editing, Resources. **Daniël L.A. van den Hove:** Funding acquisition, Conceptualization, Writing - original draft, Writing - review & editing, Supervision, Project administration. **Katie Lunnon:** Funding acquisition, Conceptualization, Writing - original draft, Writing - review & editing, Supervision, Project administration.

## Appendix A Supplementary data

Data Profile
