# Supplementary material for: An epigenome-wide association study of Alzheimer's disease blood highlights robust DNA hypermethylation in the HOXB6 gene
Source: Neurobiol Aging. 2020 Nov;95:26–45. doi: 10.1016/j.neurobiolaging.2020.06.023 (PMC7649340; doi:10.1016/j.neurobiolaging.2020.06.023)

APPENDIX B: SUPPLEMENTARY FIGURES

Supplementary Figure 1: Correlations between the first three principle components (PC1-3) in the full dataset of 284 samples, and the variables selected as covariates for further analyses: age, sex, cell type proportion (CD8T, CD4T, NK; natural killer cells, B cells, Mono; monocytes, Gran; granulocytes), and batch number (Plate1-4).

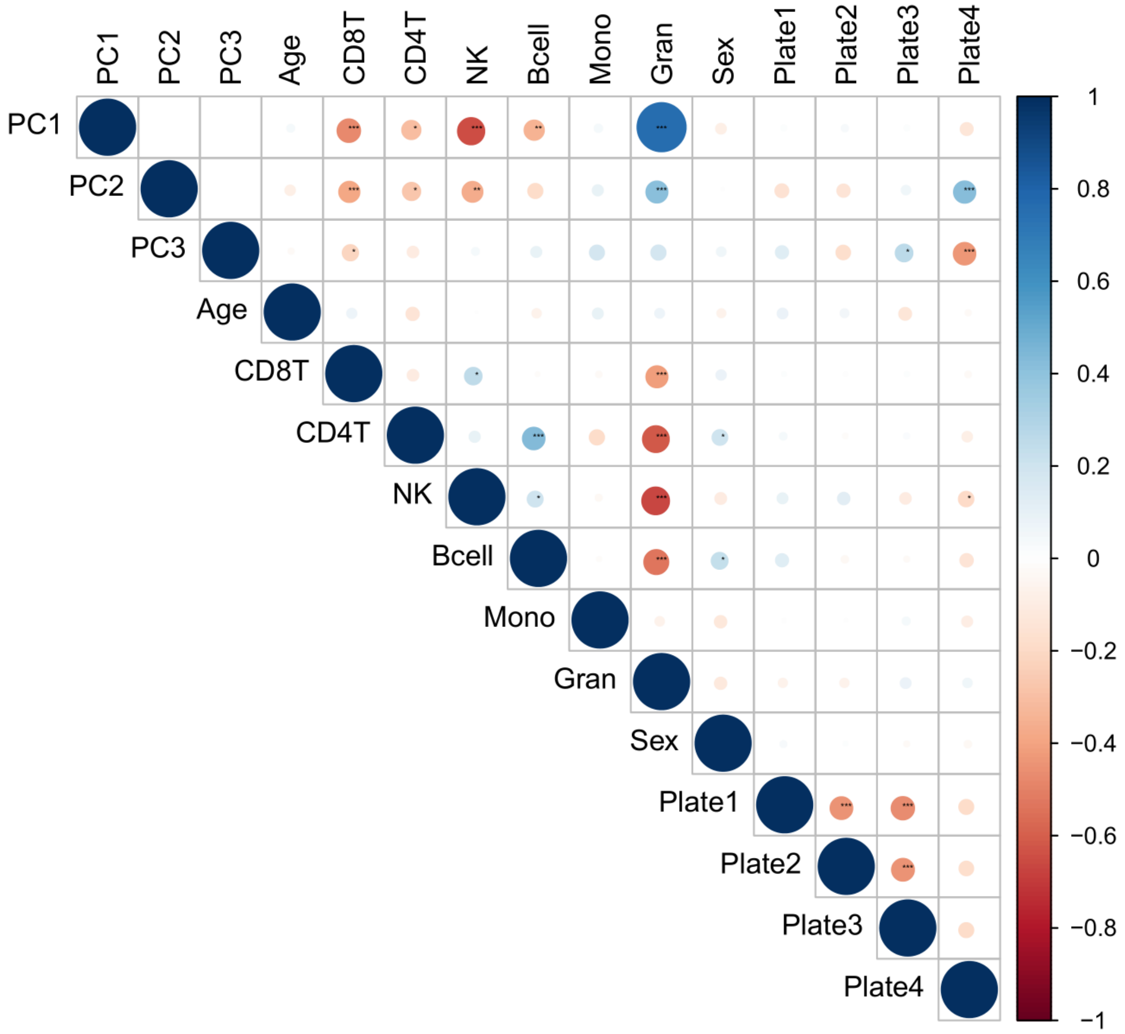

Supplementary Figure 2: Quantile-quantile (QQ) plots of (**A**) p-values from the ANOVA comparing methylation between control (CTL), mild cognitive impairment (MCI), and Alzheimer's disease (AD) samples and (**B**) p-values from the linear regression analysis comparing MCI stable (MCI-MCI) to MCI who converted to AD within one year (MCI-AD).

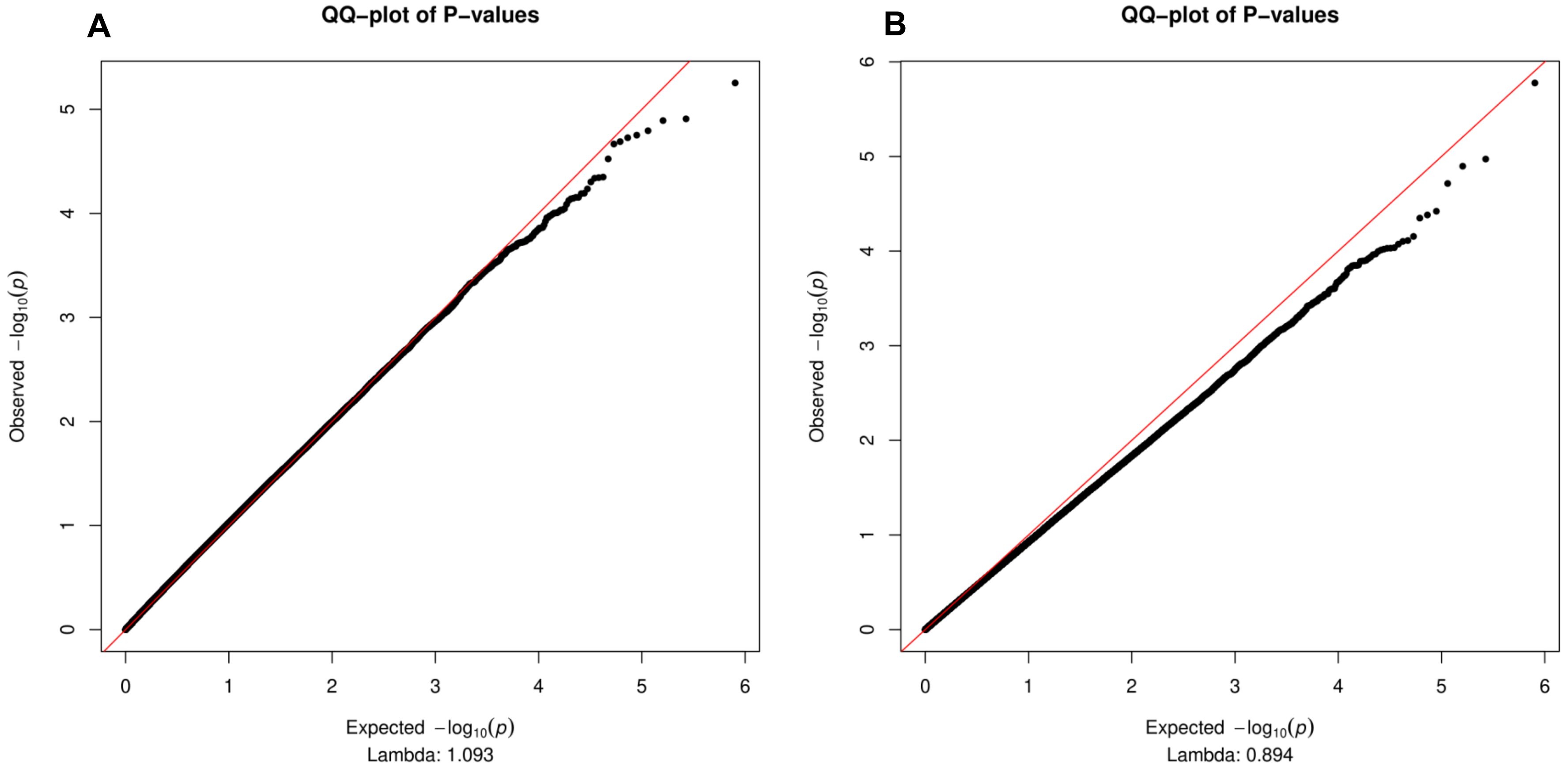

Supplementary Figure 3: (**A**) Average gene expression levels of *CSNK1E* in individuals with Alzheimer's disease (AD: yellow), mild cognitive impairment (MCI: green) or controls (CTL: blue), controlling for covariates. (**B**) Average DNA methylation levels of *CSNK1E* across its associated differentially methylated region (DMR) in AD, MCI and CTL subjects, controlling for covariates. (**C**) Correlations of DNA methylation and gene expression in *CSNK1E,* shown by disease group (blue: CTL, green: MCI, yellow: AD), and across all samples (black dotted line).

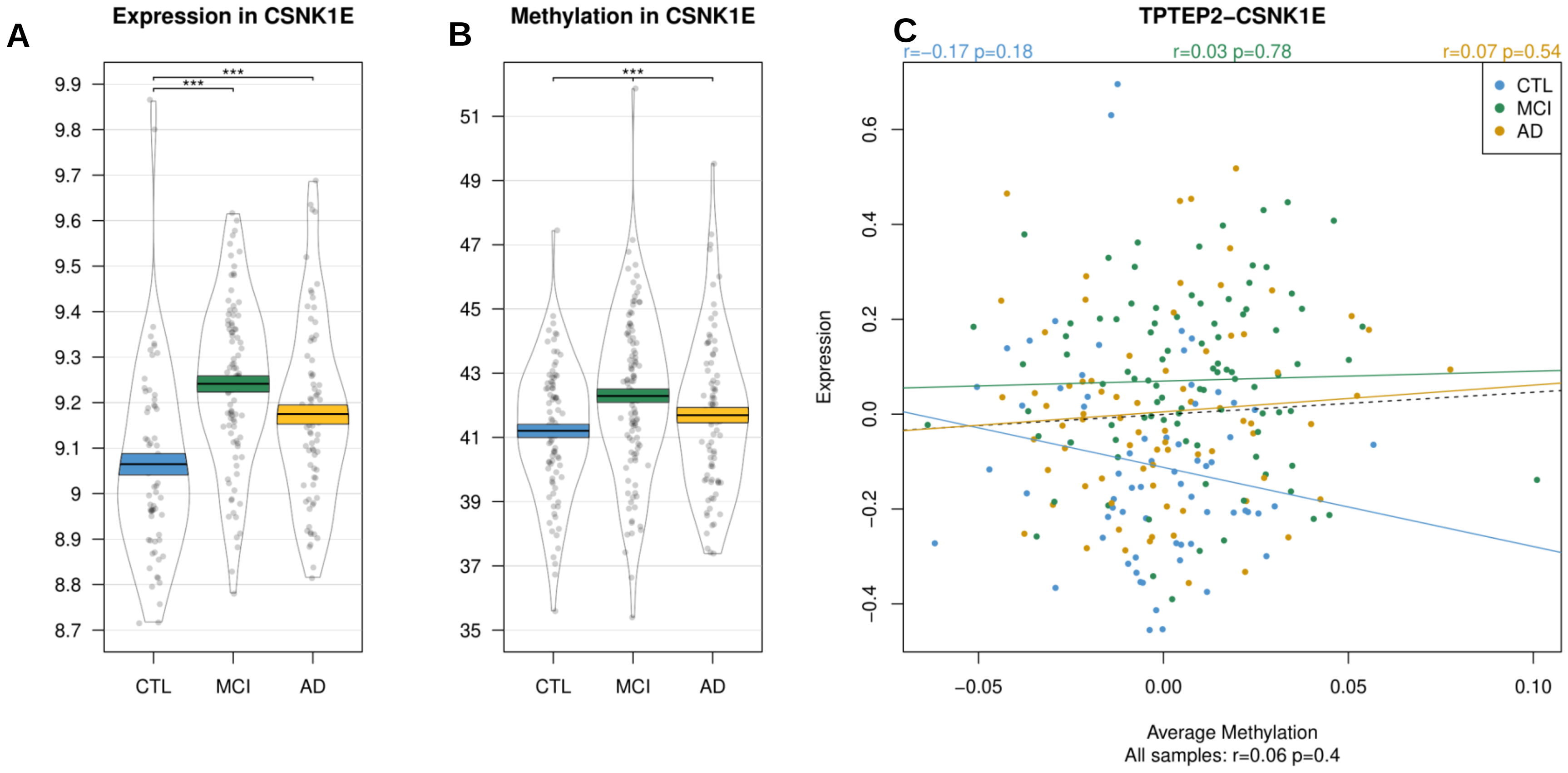

Supplementary Figure 4: (**A**) Average gene expression levels of *HOXB6* in individuals with Alzheimer's disease (AD: yellow), mild cognitive impairment (MCI: green) or controls (CTL: blue), controlling for covariates. (**B**) Average DNA methylation levels of *HOXB6* across its differentially methylated region (DMR) in AD, MCI and CTL subjects, controlling for covariates. (**C**) Correlations of DNA methylation and gene expression in *HOXB6* shown by disease group (blue: CTL, green: MCI, yellow: AD), and across all samples (black dotted line).

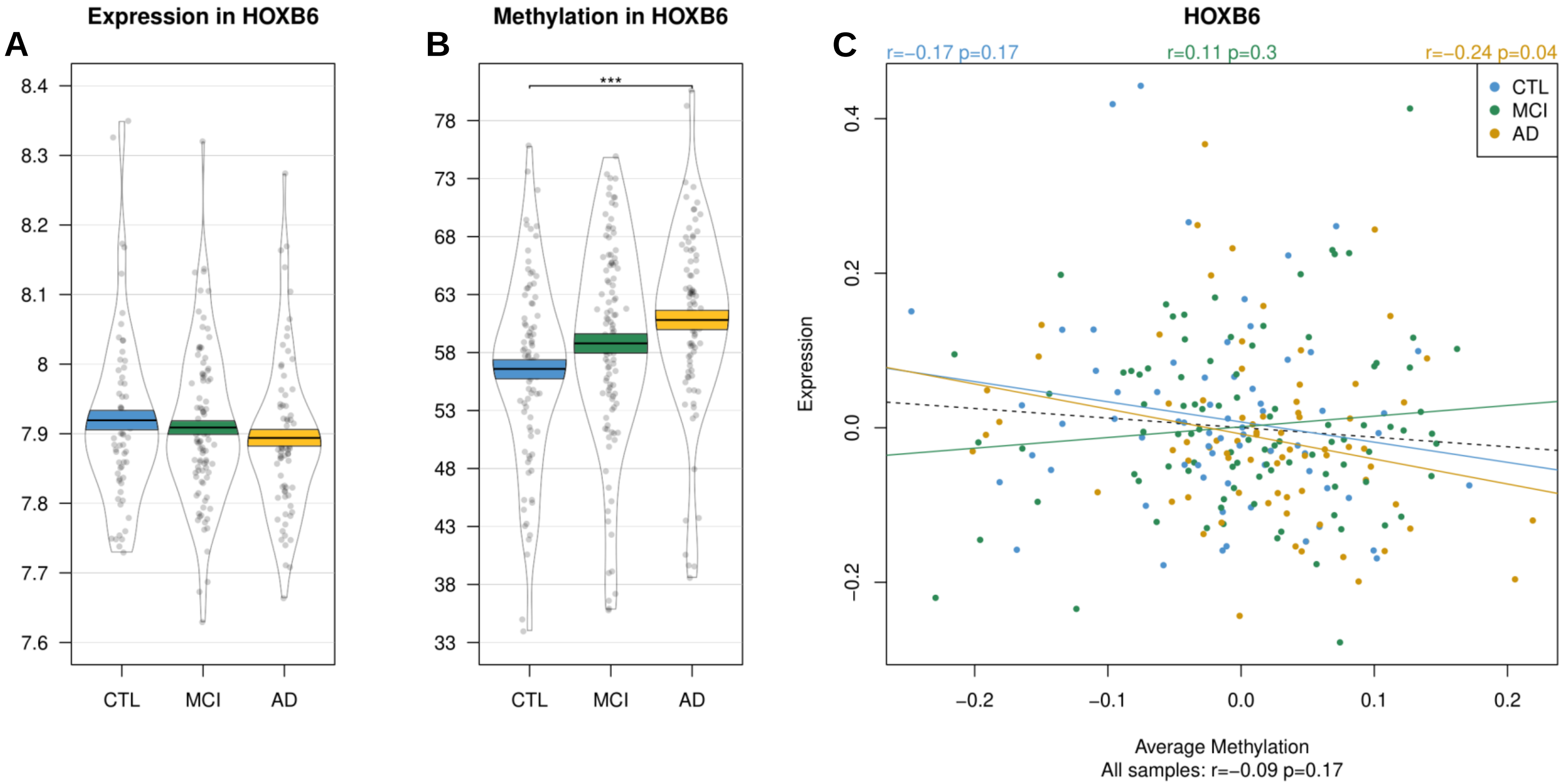

Supplementary Figure 5: Correlation of DNA methylation and gene expression in *CPT1B* shown by disease group (light green: MCI-MCI, dark green: MCI-AD), and across all samples (black dotted line). A significant correlation was observed across all samples (r=0.4, p=8.62 x 10^{-5}), which was primarily driven by a correlation observed in the MCI-MCI samples (r=0.49, p=7.27x10^{-5}) and not the MCI-AD samples.

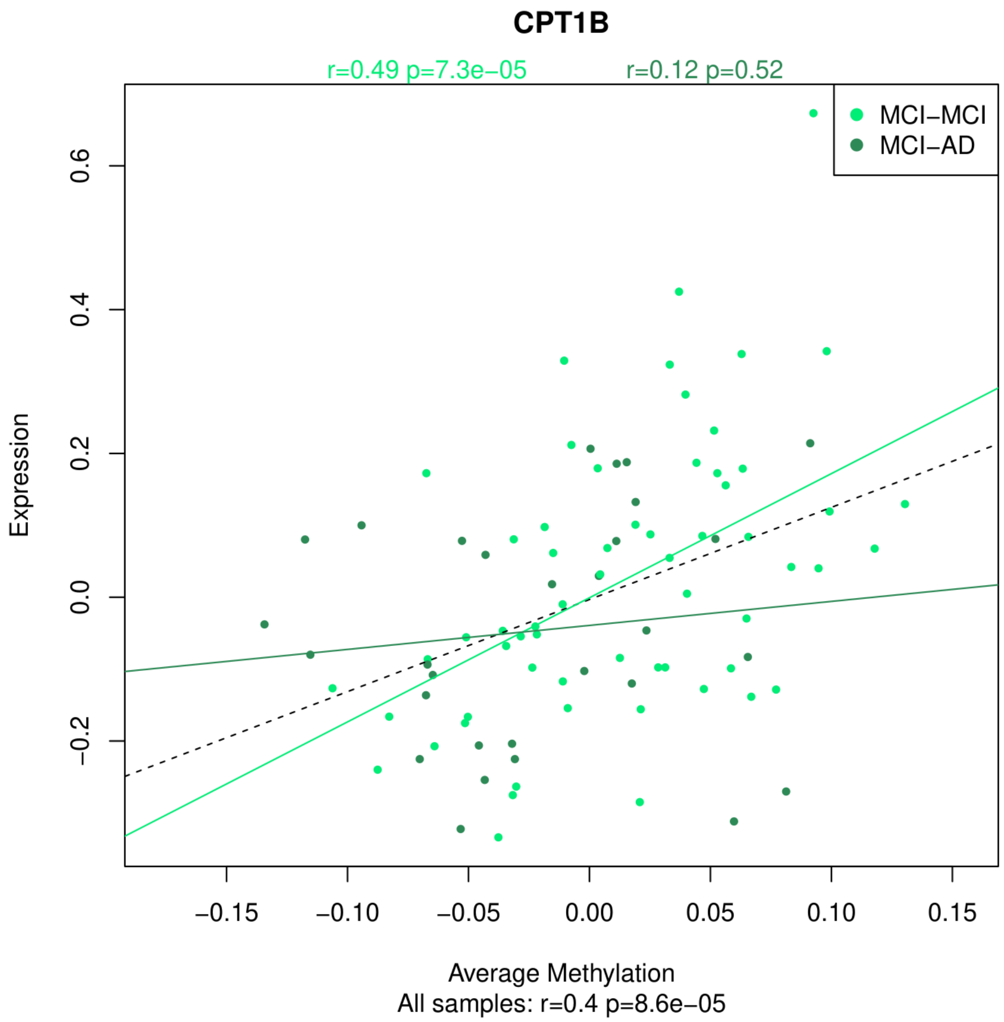

Supplementary Figure 6: Modules showing positive correlations between Module Membership (MM) and Probe Significance (PS) and that were associated with diagnosis or a trait are shown. The (**A**) brown, (**B**) purple, and (**C**) yellow modules were associated with changes in mild cognitive impairment (MCI) subjects relative to controls (CTL). (**D**) The brown module was associated with the number of education years. The (**E**) yellow module was associated with mean entorhinal thickness (MET), and (**F**) the cyan module associated with the number of *APOE ε4* alleles.

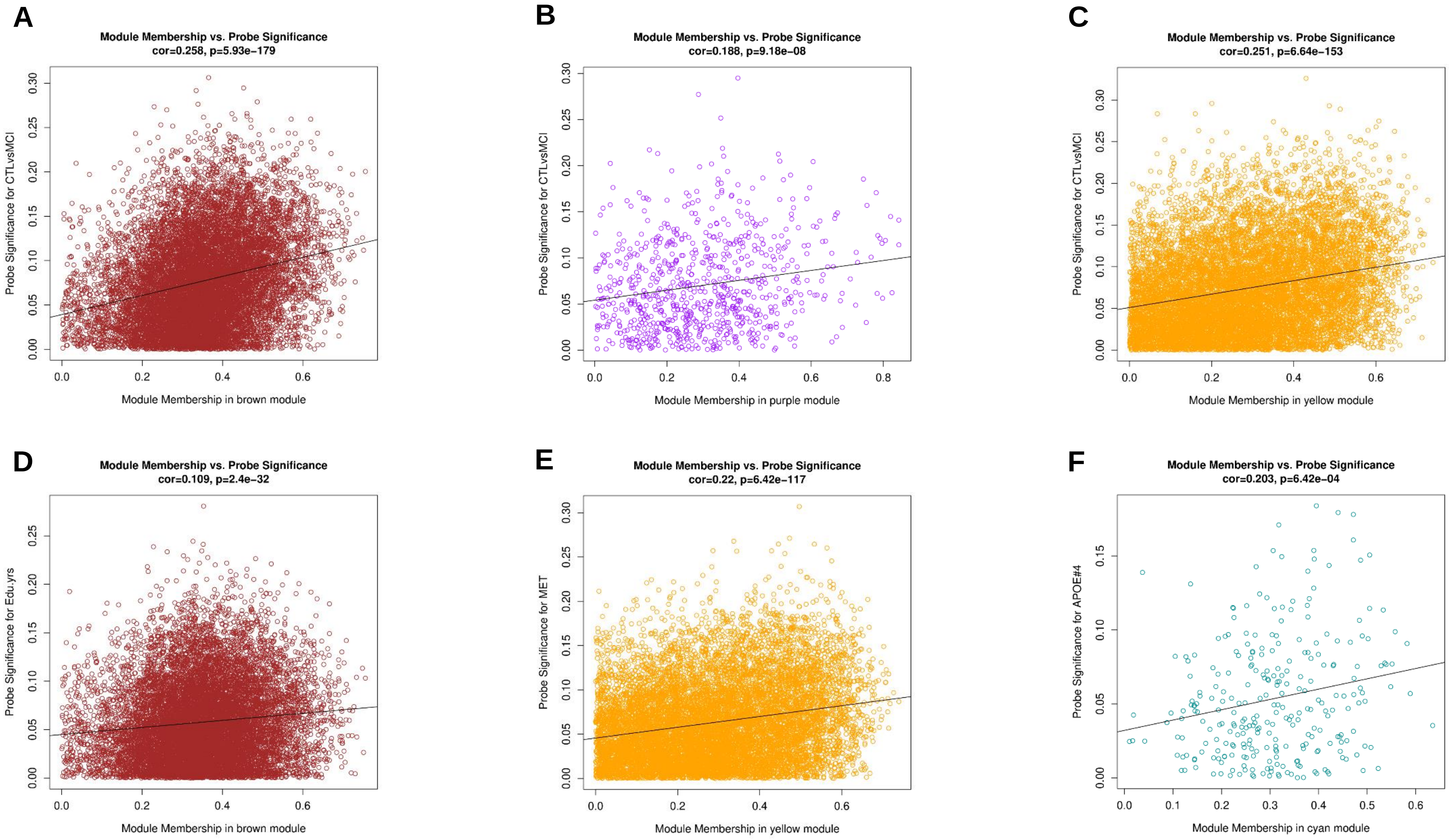

Supplementary Figure 7: (**A**) Top 20 significant Gene Ontology (GO) terms and (**B**) significant Kyoto Encyclopedia of Genes and Genomes (KEGG) terms related to the core probes within the brown module, which was associated with mild cognitive impairment (MCI) relative to controls (CTL) and number of education years. The x-axis displays the number of altered genes in the pathway.

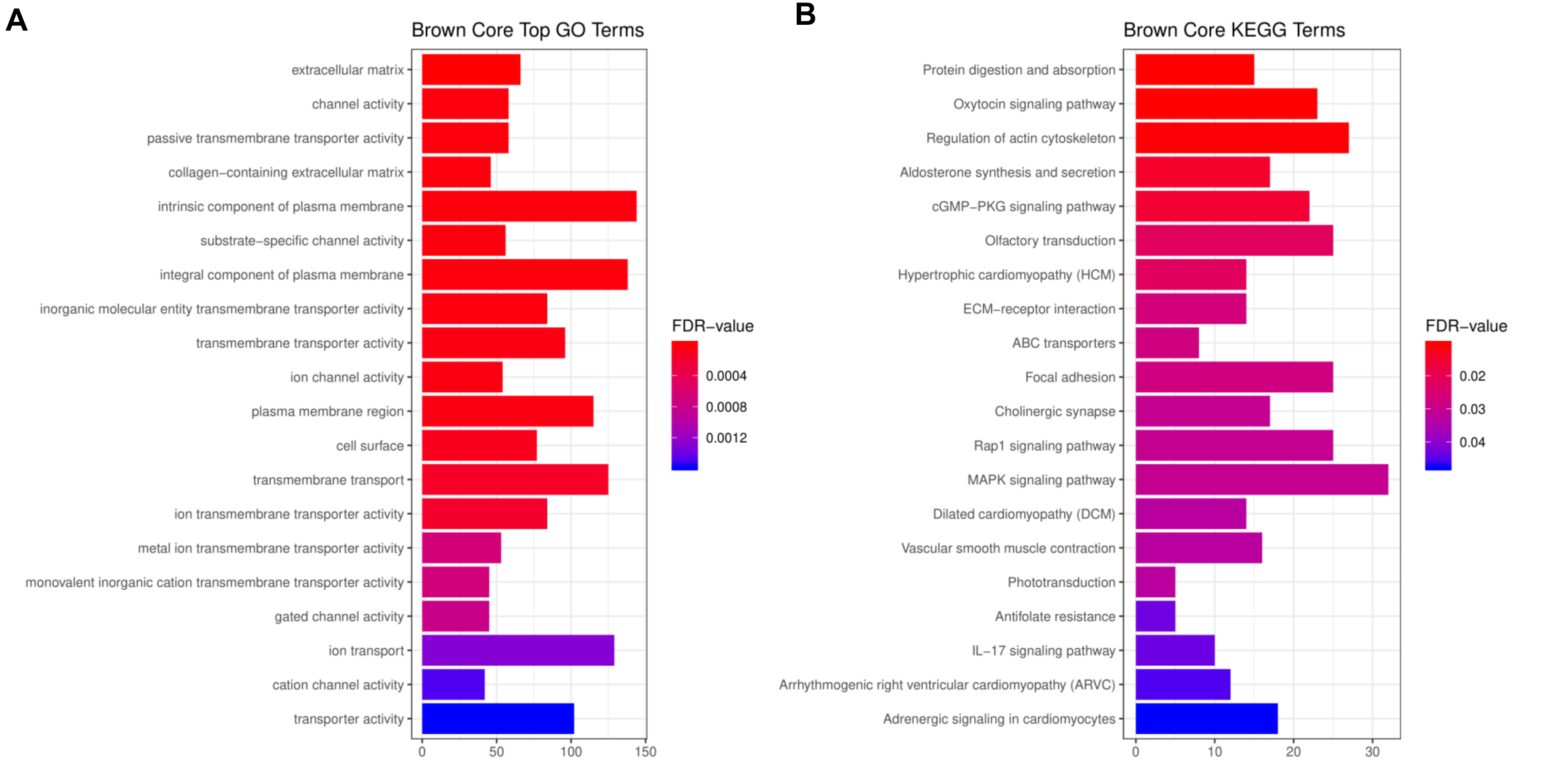

Supplementary Figure 8: (**A**) Top 20 significant Gene Ontology (GO) terms and (**B**) significant Kyoto Encyclopedia of Genes and Genomes (KEGG) terms related to the core probes within the yellow module, which was associated with mild cognitive impairment (MCI) relative to controls (CTL) and mean entorhinal thickness (MET). The x-axis displays the number of altered genes in the pathway.

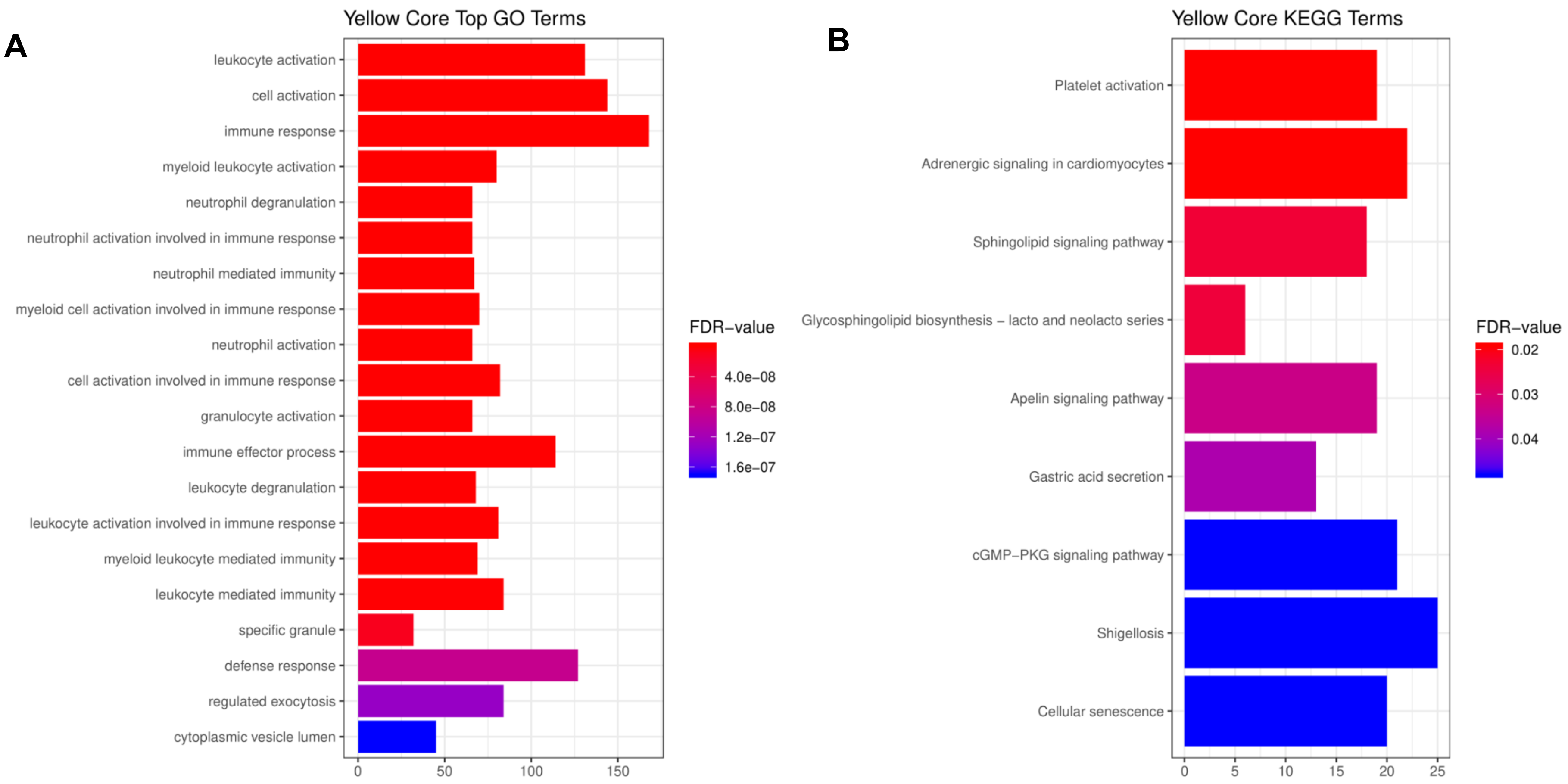

Supplementary Figure 9: Top 20 significant Gene Ontology (GO) terms related to the cyan module, which was associated with number of APOE ε4 alleles. The x-axis displays the number of altered genes in the pathway.

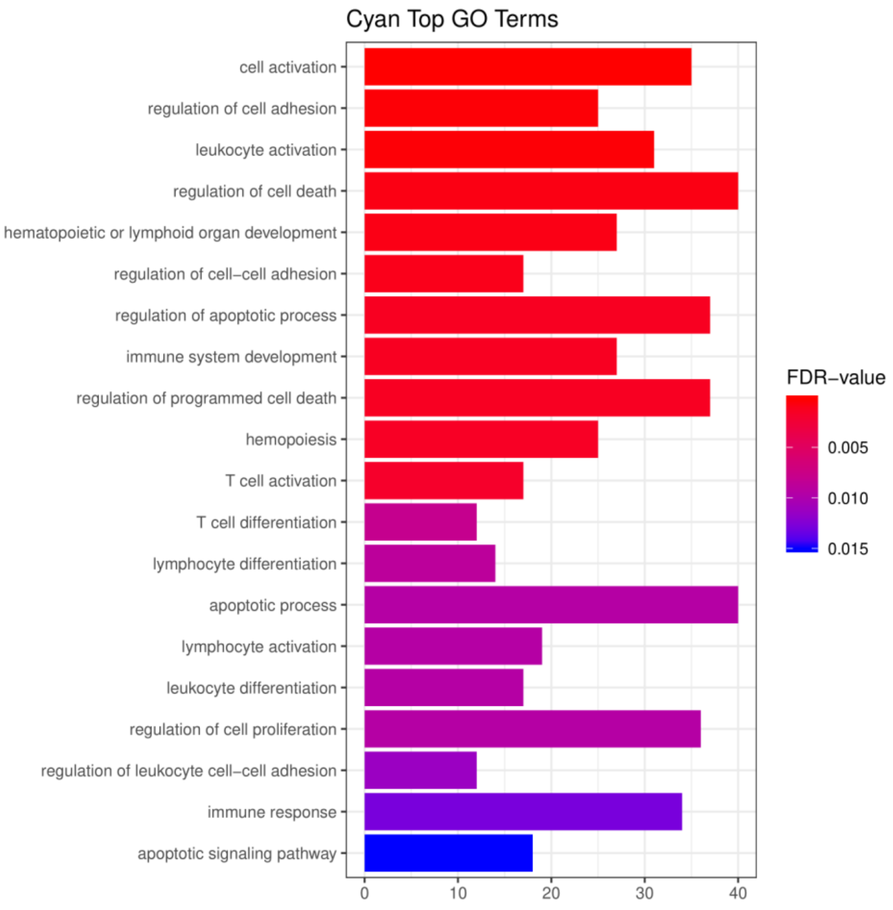

Supplementary Figure 10: Modules identified in the subset of mild cognitive impairment individuals who convert to Alzheimer's disease (MCI-AD) within one year of baseline assessment, and those who remain stable (MCI-MCI). Modules are hierarchically clustered based on calculated module eigengenes, and the number of probes included in each module are indicated along the x-axes. Colors are assigned in an arbitrary manner.

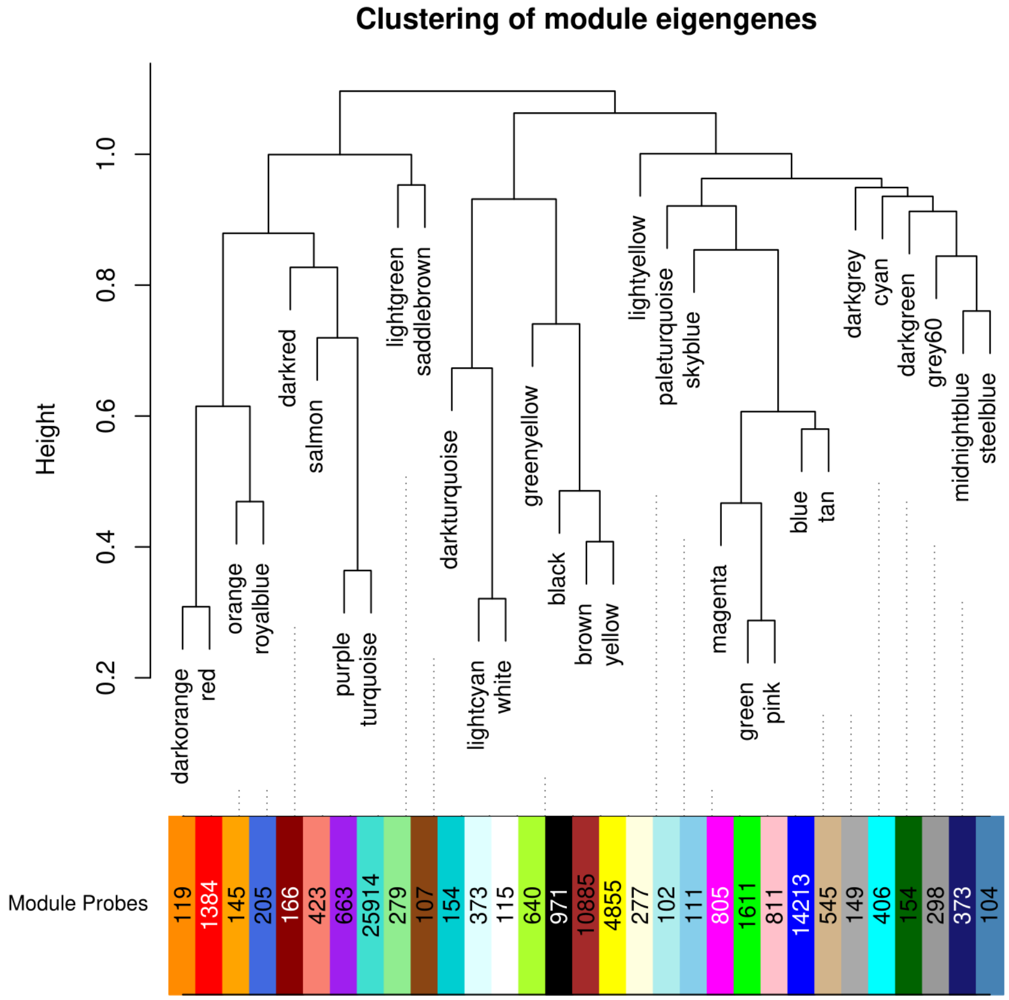

Supplementary Figure 11: Significant Kyoto Encyclopedia of Genes and Genomes (KEGG) terms related to the probes within the orange module, which was associated with conversion from MCI to AD. The x-axis displays the number of altered genes in the pathway.

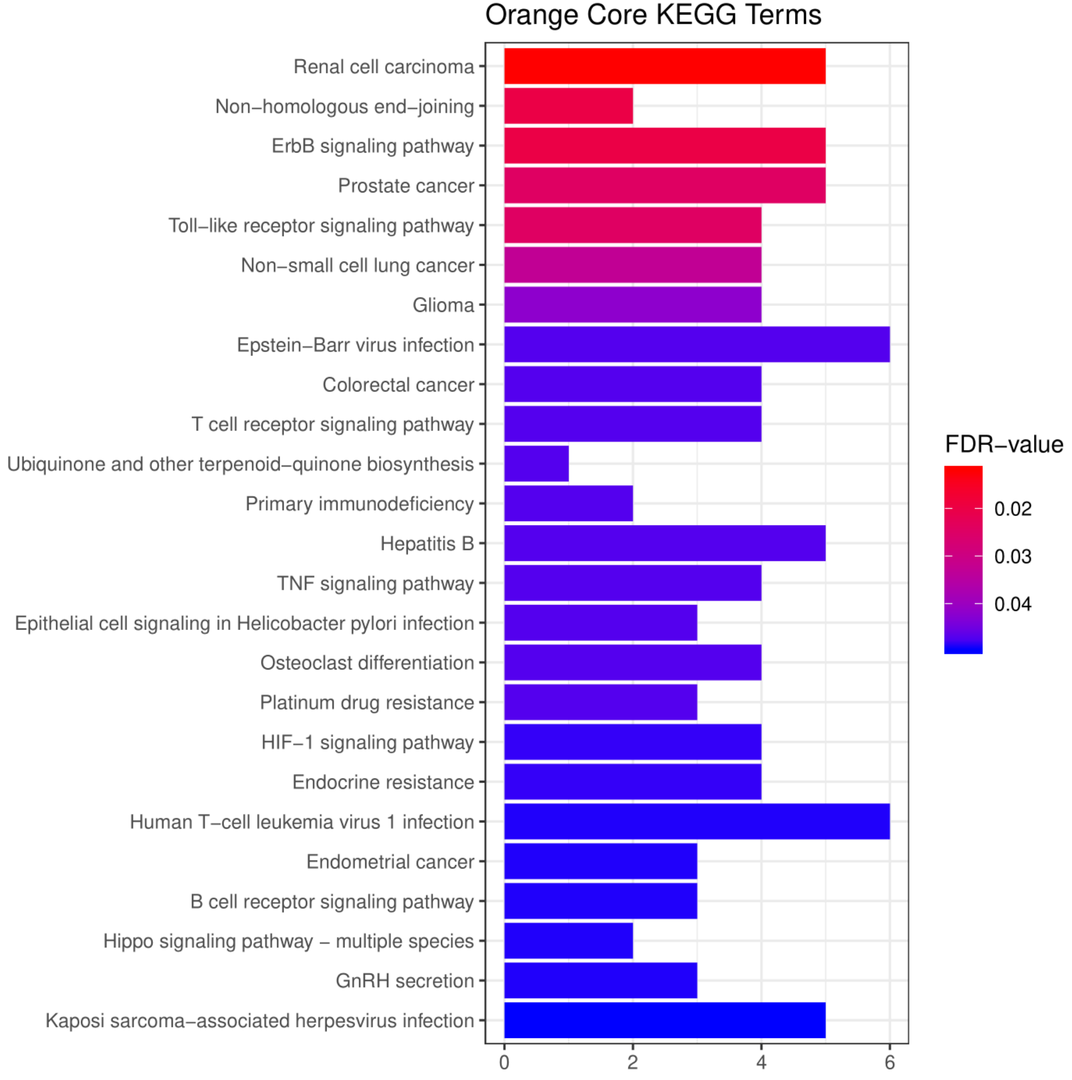

Supplement: Appendix B [file mmc3.pdf]
